# LincROR promotes tumor growth of colorectal cancer through the miR-145/WNT2B/WNT10A/Wnt/β-catenin regulatory axis

**DOI:** 10.1371/journal.pone.0312417

**Published:** 2024-11-15

**Authors:** Li-Qiang Deng, Shi-Ying Li, Tian Xie, Wei-Qiang Zeng, Yu-Yan Wang, Chuan-Jian Shi, Zhang Jin-Fang

**Affiliations:** 1 Shenzhen Traditional Chinese Medicine Oncology Center, Shenzhen, Guangdong, P. R. China; 2 Shenzhen Hospital (Futian) of Guangzhou University of Chinese Medicine, Shenzhen, Guangdong, P. R. China; 3 Guangdong Provincial Key Laboratory of New Drug Screening, School of Pharmaceutical Sciences, Southern Medical University, Guangzhou, P. R. China; Università degli Studi della Campania, ITALY

## Abstract

Colorectal cancer (CRC) is a prevalent form of malignant tumor, and the current clinical treatments are far from satisfactory. Identifying new therapeutic targets is therefore essential for clinical practices. The long intergenic non-protein coding RNA lincROR has been shown to play a significant role in the tumorigenesis of various cancers. However, the molecular mechanism underlying lincROR-mediated CRC tumorigenesis remains unclear. In the present study, we found that knockdown of lincROR significantly inhibited cell viability *in vitro*, while its overexpression promoted tumor growth *in vivo*. Mechanistically, lincROR acted as a miRNA sponge for miR-145, thereby elevating the expression of the target genes WNT2B and WNT10A. The overexpression of WNT2B and WNT10A definitely activated the Wnt/β-catenin pathway, thus led to promoting tumorigenesis in CRC. In summary, our findings identified lincROR as a novel activator of the Wnt/β-catenin pathway by serving as a miRNA sponge for miR-145 and facilitating tumorigenesis, which suggests that lincROR may be a potential therapeutic target for CRC patients.

## Introduction

Colorectal Cancer (CRC) is the third most common and second largest malignant cancer, which has become one of the most pressing public health problems worldwide [1]. With the improvement in therapeutic techniques, the survival rate of CRC has been significantly increased. However, the prognosis of CRC remains poor because most patients are diagnosed at advanced stages. Therefore, there is a significant need to identify early diagnostic biomarkers or new therapeutic targets for CRC patients.

Long noncoding RNAs (lncRNAs) are a class of transcripts without protein-coding ability. Increasing evidence has demonstrated that lncRNAs play significant roles in various biological activities and disease progression. As a member of ncRNA family, long intergenic noncoding RNA ROR (lincROR) was initially reported to mediate the reprogramming and transformation of human induced pluripotent stem cells (iPSCs) and interfere with the specific differentiation of embryonic stem cells (ESCs) [2]. Recent studies showed that lincROR was obviously up-regulated in various tumors, including colorectal cancer, breast cancer, pancreatic cancer, hepatocellular carcinoma, and lung cancer [3]. For instance, lincROR enhanced the proliferation, invasion, and migration of gastric cancer cells *via* the miR-212-3p/FGF7 axis [4]. Our previous study also reported that curcumin inhibited the proliferation of hepatocellular carcinoma cells by suppressing lincROR expression and inactivating Wnt/β-catenin signalling [5]. Considering that Wnt/β-catenin signalling is closely associated with CRC tumor progression and tumorigenesis, we justly wondered whether lincROR mediated the Wnt/β-catenin signalling in CRC tumorigenesis.

In the present study, lincROR was found to promote CRC tumorigenesis *in vitro* and *in vivo*. Serving as a competing endogenous RNA (CeRNA), lincROR directly targeted miR-145 and suppressed its expression. Moreover, miR-145 was identified to suppress the expression of target genes WNT2B and WNT10A, which induced the inactivation of Wnt/β-catenin signalling and inhibited cell viability in CRC cells. Collectively, our results showed that lincROR acted as a miRNA sponge for miR-145 and de-repressed the expression of WNT2B and WNT10A, which led to activating Wnt/β-catenin signalling pathway and promoting tumorigenesis in CRC.

## Results

### Knockdown of lincROR suppressed cell viability and induced the inactivation of Wnt/β-catenin signalling in CRC cells

**Fig 1:**
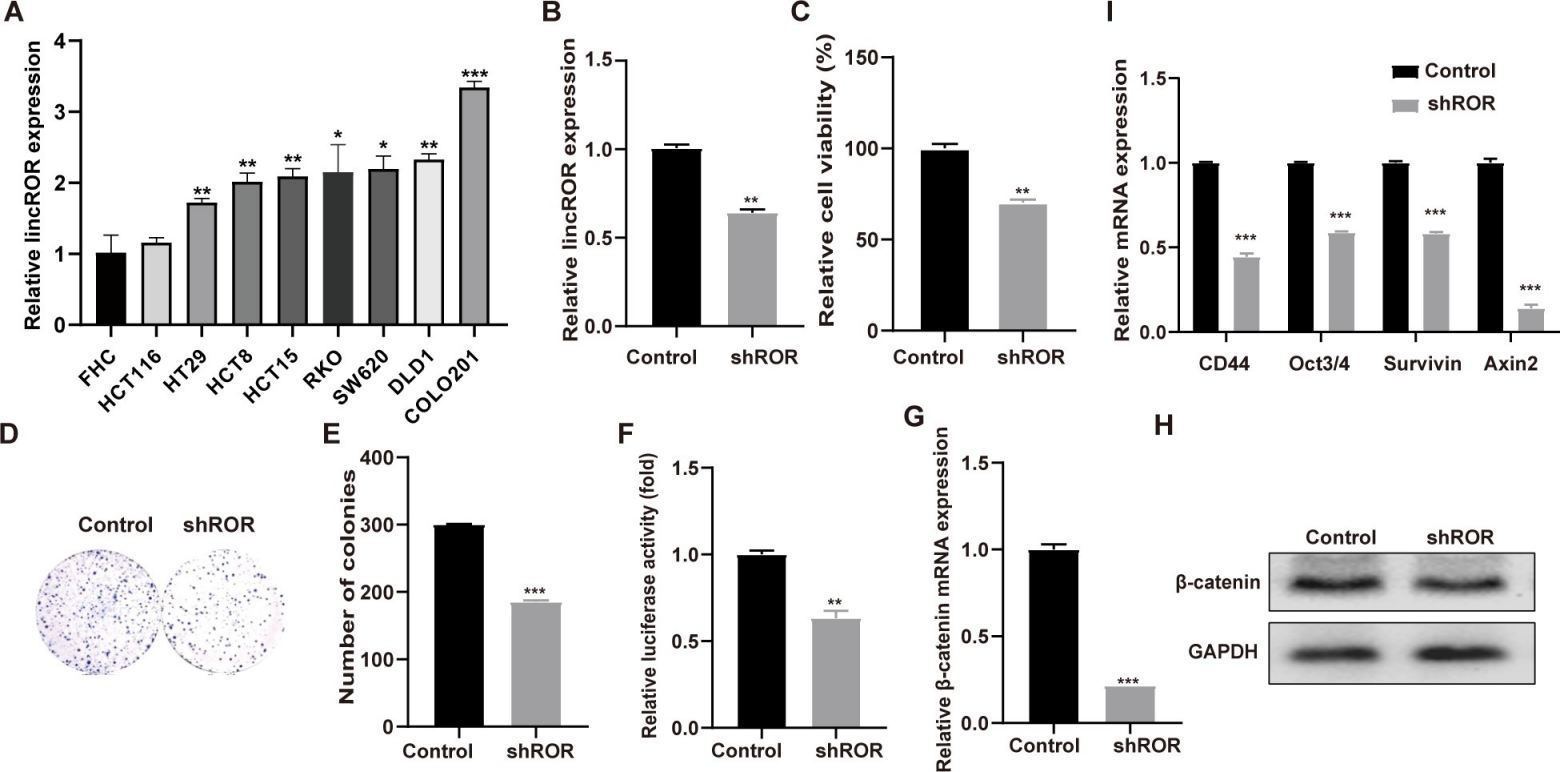
Knockdown of lincROR suppressed the cell viability and induced the inactivation of Wnt/β-catenin signalling in CRC cells. **(A),** The lincROR expression was examined in a panel of CRC cells. **(B),** LincROR expression was suppressed in SW62 cells infected with shROR. **(C-E),** Cell viability **(C)** and colony formation **(D, E)** were assessed in the lincROR knockdown cells. **(F),** The TOP flash luciferase activity was examined in the lincROR knockdown cells using dual luciferase assays. **(G-H),** β-catenin expression was determined in the lincROR silenced SW620 cells by Western blotting and qRT-PCR analysis. **(I),** The expression of several downstream targets of Wnt/β-catenin pathway was analysed in lincROR knockdown cells through qRT-PCR examination. Data were shown as mean ± SD (n = 3). *, *P* < 0.05; **, *P* < 0.01; ***, *P* < 0.001; versus the corresponding control group.

LincROR has been reported to be upregulated and to act as an oncogene in several cancers [6], we therefore examined its expression in a panel of CRC cell lines. The results showed that it was significantly upregulated in most CRC cells compared to the normal human fetal colon cell line FHC (Fig 1A), suggesting that upregulation of lincROR might be a common phenomenon in CRC. To further investigate the function of lincROR in CRC, a specific lincROR knockdown plasmid was generated (shROR) and it was found that the expression of lincROR was significantly suppressed by shROR in SW620 cells (Fig 1B). We also found that cell viability and colony formation ability were obviously inhibited by lincROR knockdown in SW620 cells (Fig 1C–1E). Aberrant activation of the Wnt/β-catenin signalling pathway has been reported in colorectal cancer, and our previous study also demonstrated that lincROR activated the Wnt/β-catenin signalling pathway during pathogenesis of mesenchymal stem cells [7]. We therefore wondered whether this signalling cascade was involved in lincROR-mediated tumorigenesis. As shown in Fig 1F, the luciferase activity of the Wnt signaling reporter TOPflash was significantly suppressed by lincROR knockdown in SW620 cells. Consistently, the crucial transcription factor β-catenin was found to be suppressed at mRNA and protein levels by the silence of lincROR (Fig 1G and 1H). In addition, the RNA expression levels of several downstream effectors of the Wnt/β-catenin signalling pathway, including CD44, Oct3/4, Survivin, and Axin2, were significantly decreased with lincROR knockdown (Fig 1I).

### LincROR directly targeted miR-145 and miR-145 negatively regulated CRC cells growth and Wnt/β-catenin signalling

**Fig 2:**
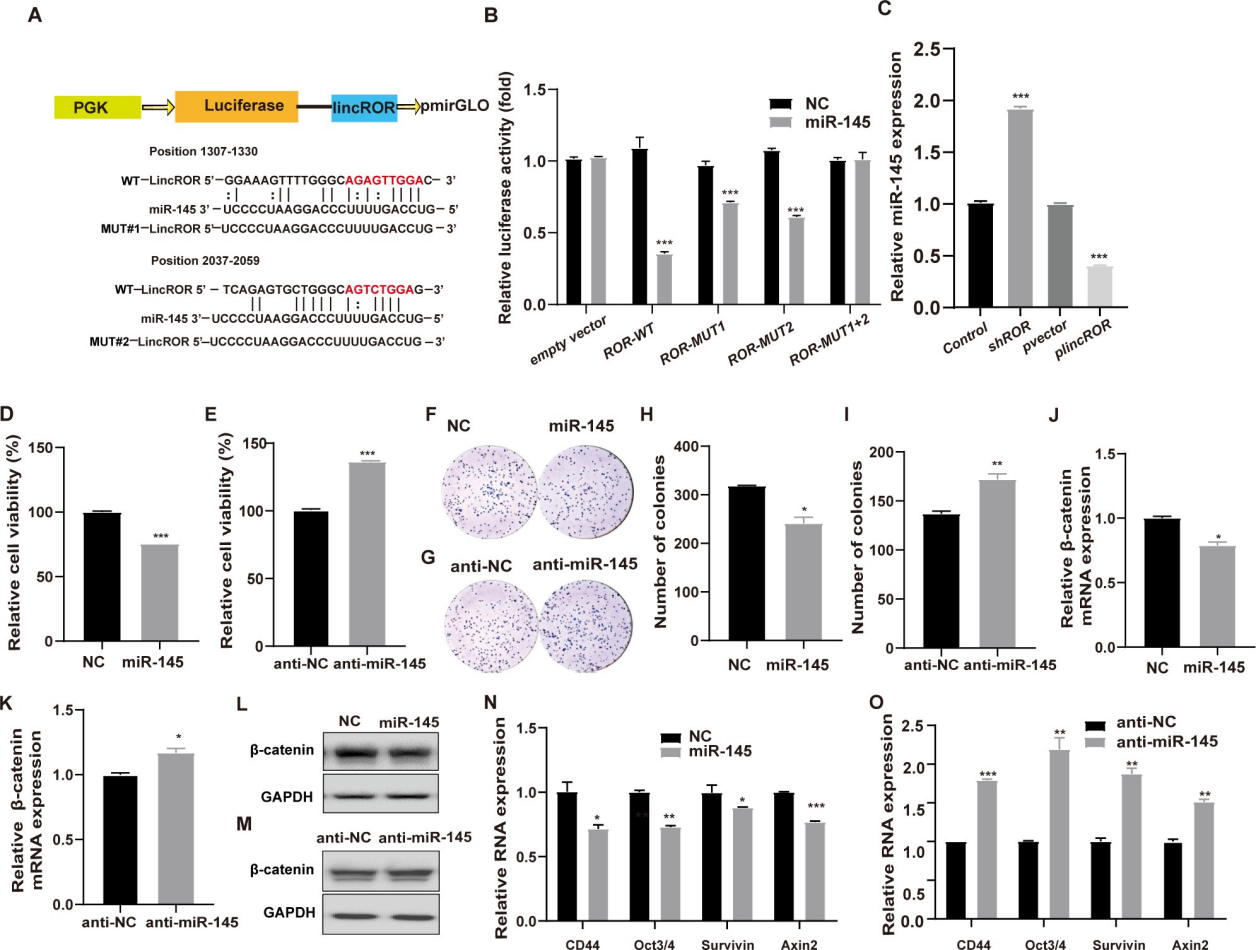
LincROR-targeted MiR-145 acted as a negative regulator of CRC cells growth *via* suppressing Wnt/β-catenin signalling. **(A),** The predicted binding sites of miR-145 in lincROR were shown in red, and the binding sequence was inserted into the pmirGLO vector. **(B),** The CRC cells were co-transfected with miR-145 mimics and WT or MUT luciferase reporter, and the luciferase activity was measured by dual luciferase reporter assay. **(C),** The miR-145 expression was measured by qRT-PCR examination with lincROR overexpression or knockdown. **(D-E),** The cell viability was evaluated by MTT assay in miR-145 mimics or anti-miR-145 transfected SW620 cells. **(F-I),** the colony formation was examined in miR-145 mimics or anti-miR-145 transfected CRC cells. **(J-M),** The mRNA and protein levels of β-catenin were examined in CRC cells with miR-145 or anti-miR-145 transfection. **(N-O),** The relative expression of several downstream target genes of the Wnt/β-catenin pathway were examined by qRT-PCR in the miR-145 or anti-miR-145 transfected SW620 cells. Data were shown as mean ± SD (n = 3). *, *P* < 0.05; **, *P* < 0.01; ***, *P* < 0.001; versus the corresponding control group.

MicroRNA-145 (miR-145) was predicted to directly bind lincROR by bioinformatics analysis and we constructed the wild and mutated luciferase reporters according to the direct binding sequences (Fig 2A). The luciferase activity assays demonstrated that miR-145 significantly reduced the luciferase activity of wild type (WT) reporter, whereas a deleted mutation type (MUT) successfully abolished this suppressive effect (Fig 2B). Furthermore, miR-145 expression was upregulated by lincROR knockdown while it was downregulated by lincROR overexpression (Fig 2C). To explore the biological function of miR-145 in CRC, cell viability was examined after transfected with miR-145 mimics and inhibitors. The results showed that miR-145 mimics significantly inhibited while anti-miR-145 oligoes facilitated cell viability in CRC cells (Fig 2D and 2E). The further colony formation assays also confirmed the tumor-suppressive role of miR-145 in CRC cells (Fig 2F–2I). To clarify the mechanism underlying the miR-145-induced cell growth suppression, cell cycle and apoptosis were examined by the flow cytometry examination. As shown in S1A Fig, the cell cycle analysis revealed that more cells were arrested at G0/G1 phase in the miR-145 transfected SW620 cells. Subsequent apoptosis assays indicated that the number of apoptotic cells was significantly increased following transfection with miR-145 in SW620 cells (S1B Fig). These results suggested that miR-145 suppressed CRC cell growth *via* inducing both cell cycle arrest and apoptosis. We next investigated whether miR-145 mediated the Wnt/β-catenin pathway in CRC cells. The results showed that miR-145 mimics obviously suppressed while anti-miR-145 enhanced the expression of β-catenin at both mRNA and protein levels (Fig 2J–2M). Furthermore, the RNA levels of several downstream transcriptional targets such as CD44, Oct3/4, Survivin and Axin2 were decreased by miR-145 whereas they were increased by anti-miR-145 (Fig 2N and 2O), indicating that miR-145 suppressed the activation of Wnt/β-catenin pathway in CRC cells.

### WNT2B and WNT10A were common targets for miR-145

**Fig 3:**
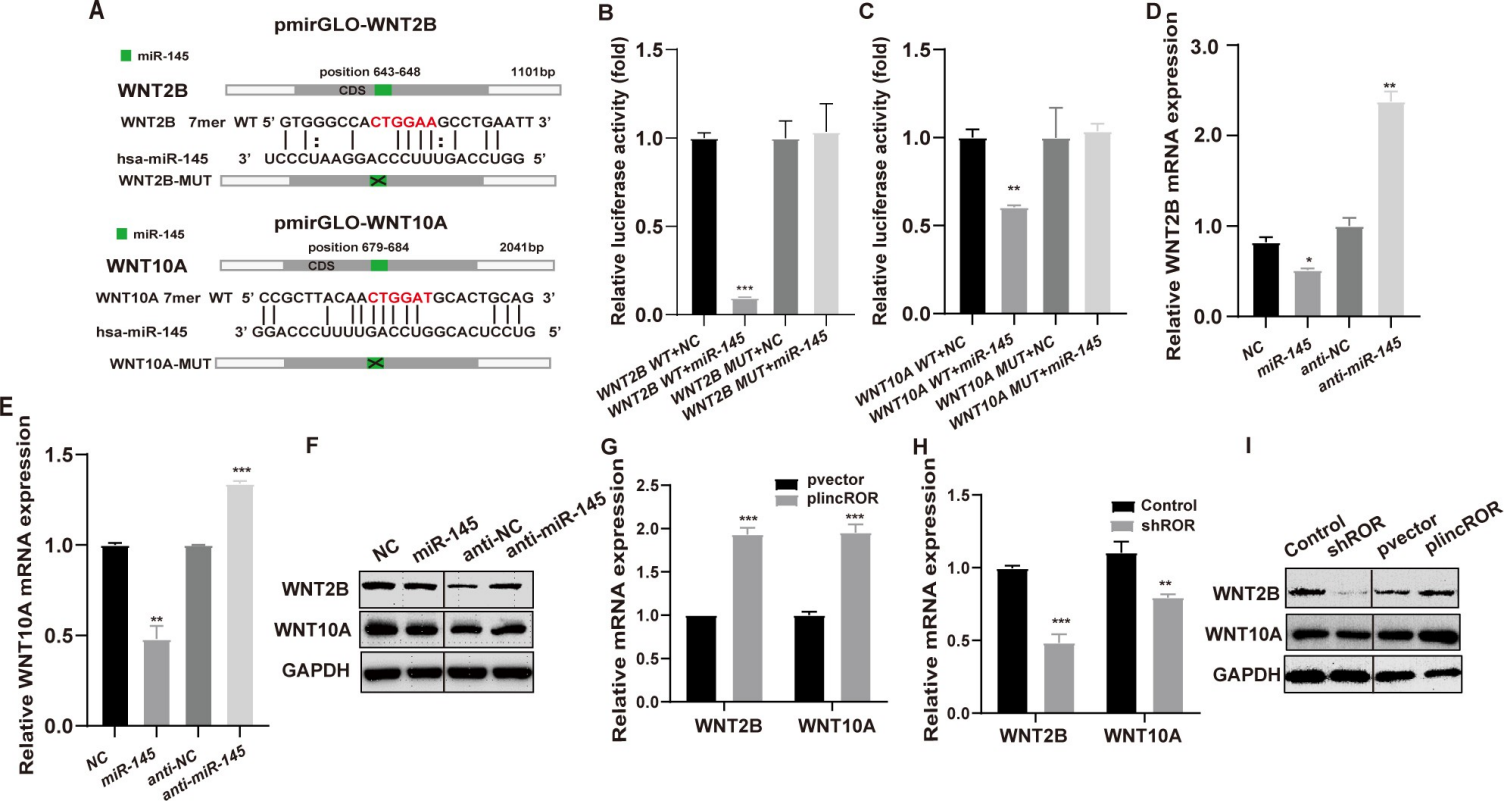
WNT2B and WNT10A were real targets of miR-145 in CRC cells. **(A),** The binding sequence of miR-145 in WNT2B/WNT10A 3’UTR was inserted into the dual-luciferase reporter vector to generate the WT plasmids. In contrast, this binding sequence was deleted to generate MUT plasmids. **(B-C),** Following transfection with miR-145, the luciferase activities of WT and MUT reporters were assessed. **(D-F),** The mRNA and protein expression levels of WNT2B and WNT10A were detected in CRC cells with miR-145 or anti-miR-145 transfection into SW620 cells. **(G-I)** The mRNA and protein expression levels of WNT2B and WNT10A were examined by qRT-PCR and western blot examination in lincROR silencing or overexpressing SW620 cells. Data were shown as mean ± SD (n = 3). *, *P* < 0.05; **, *P* < 0.01; ***, *P* < 0.001; versus the corresponding control group.

It is well known that miRNAs exert their biological roles by inhibiting the expression of protein-coding genes and thereby regulating related signalling pathways. WNT2B and WNT10A were predicted as the potential targets of miR-145 by bioinformatics analysis and the luciferase reporters with wild type (WT) and Mutated binding sites (MUT) were constructed according to the binding sequence (Fig 3A). The results of luciferase activity showed that miR-145 dramatically suppressed the luciferase activity of the two WT reporters while mutations on these binding sites successfully abolished these suppressive effects (Fig 3B and 3C). Moreover, the RNA and protein levels of WNT2B and WNT10A were suppressed by miR-145 mimics transfection, whereas anti-miR-145 oligoes promoted their expression (Fig 3D–3F). Furthermore, overexpression of lincROR promoted WNT2B and WNT10A expression while knockdown of lincROR suppressed their expression at both RNA and protein levels (Fig 3G–3I). Collectively, these data demonstrated that lincROR acted as a miRNA sponge for miR-145 and de-repressed the expression of WNT2B and WNT10A.

### LincROR activated the Wnt/β-catenin signalling by interacting with miR-145

**Fig 4:**
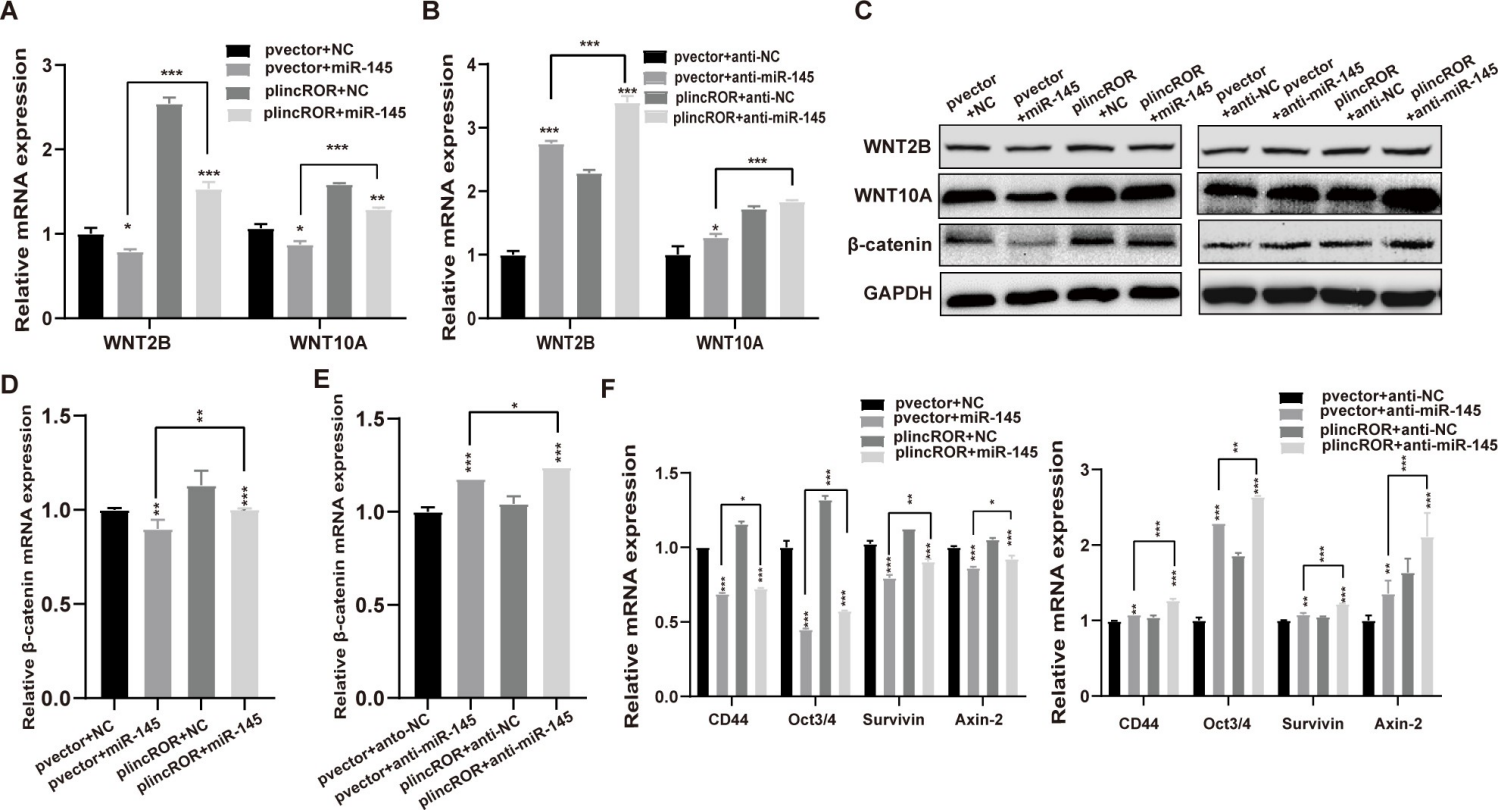
LincROR activated the Wnt/β-Catenin signalling pathway by targeting miR-145. **(A-B),** the mRNA expression levels of WNT2B and WNT10A were examined in the lincROR overexpressing SW620 cells with miR-145 or anti-miR-145 transfection. **(C),** The protein expression levels of β-catenin, WNT2B, and WNT10A were examined in the lincROR overexpressing SW620 cells with miR-145 or anti-miR-145 transfection. **(D-E),** The mRNA expression level of β-catenin was assessed in the lincROR overexpressing SW620 cells with miR-145 or anti-miR-145 transfection. **F,** The mRNA expression levels of several downstream targets of Wnt/β-catenin signalling were examined in the lincROR overexpressing SW620 cells with miR-145 or anti-miR-145 transfection. Data were shown as mean ± SD (n = 3). *, *P* < 0.05; **, *P* < 0.01; ***, *P* < 0.001; versus the corresponding control group.

To validate the crucial role of miR-145 in the lincROR-mediated the Wnt/β-catenin signalling, a rescue study was performed using SW620 cells overexpressing lincROR. As shown in Fig 4A and 4C, the decreased expression levels of WNT2B and WNT10A following miR-145 overexpression were rescued after transfection of lincROR overexpression plasmid. Moreover, lincROR overexpression enhanced the up-regulation of two target genes induced by transfection with anti-miR-145 (Fig 4B and 4C). Consistent results were observed in β-catenin expression in lincROR overexpressing SW620 cells after transfected with miR-145 mimics or anti-miR-145 (Fig 4C–4E). Furthermore, the forced expression of lincROR successfully restored the decreased expression of several downstream targets of the Wnt/β-catenin pathway induced by miR-145 mimics, whereas lincROR overexpression partially aggravated their enhanced expression induced by anti-miR-145 (Fig 4F). Collectively, these findings indicated that lincROR increased the expression of WNT2B and WNT10A, thereby activating Wnt/β-catenin signaling in CRC cells.

### LincROR facilitated the tumorigenesis of CRC *in vivo*

**Fig 5:**
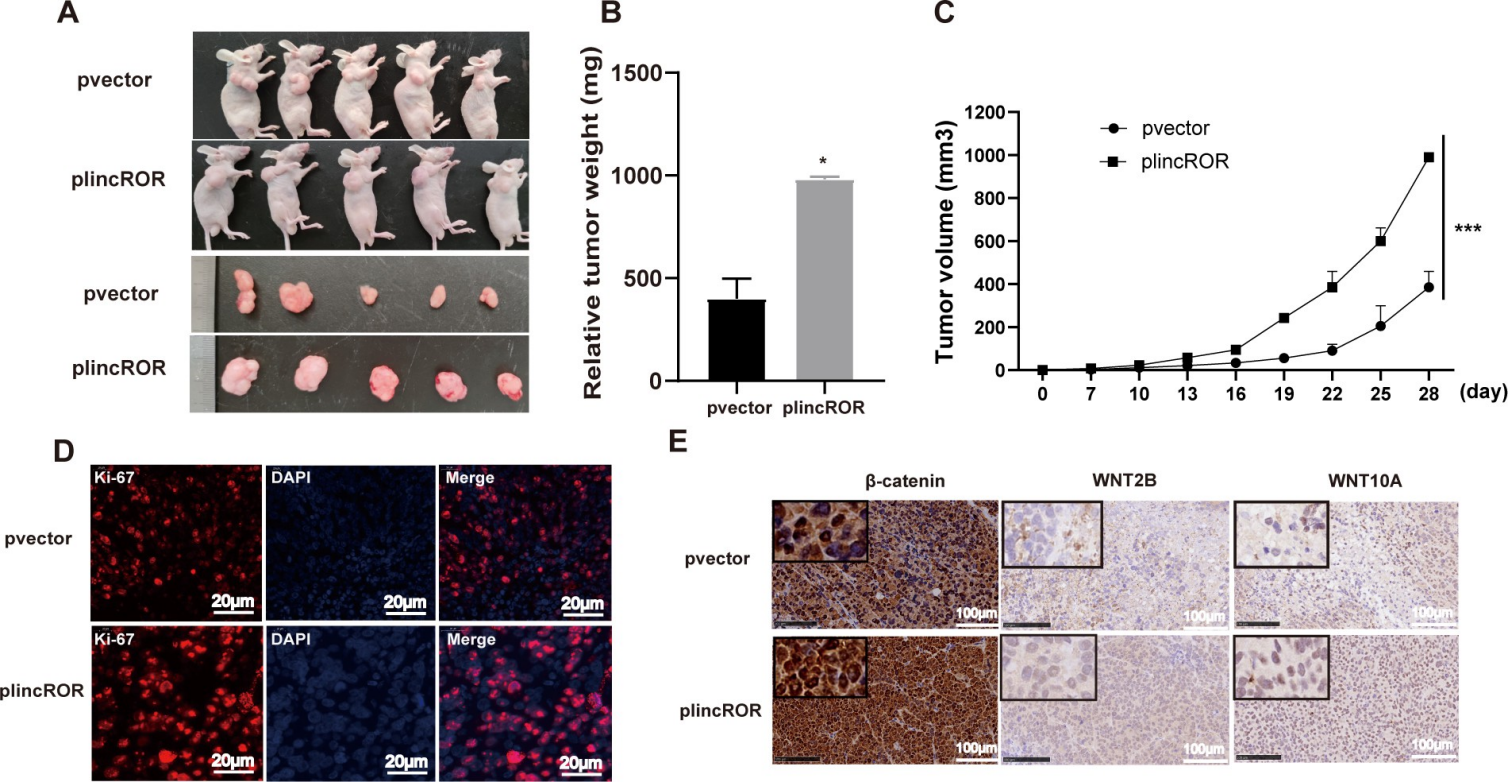
LincROR overexpression promoted the tumor growth of CRC in *vivo*. **(A),** Representative images of burdened tumors in nude mice (n = 5). **(B-C),** Tumor weights and volumes were analyzed in the lincROR overexpression group and the control group. **(D),** The immunofluorescence analysis of Ki-67 was performed on tumor tissues (Scale bar, 20 μm.). **(E),** The immunohistochemical analysis of β-catenin, WNT2B, and WNT10A was conducted on tumor tissues (Scale bar, 100 μm.). Data were shown as mean ± SD (n = 5). *, *P* < 0.05; ***, *P* < 0.001; vs NC; versus the corresponding control group.

To further examine the functional characterization of lincROR in tumorigenesis, the stable lincROR-overexpressing SW620 cells were subcutaneously injected into flanks of nude mice to establish the xenograft tumor model. The overexpression of lincROR significantly promoted tumor growth *in vivo*, as evidenced by the increased tumor volume and weight (Fig 5A–5C). The further immunofluorescence analysis revealed that lincROR promoted the expression of cell proliferation marker Ki67 (Fig 5D). Additionally, the levels of β-catenin, WNT2B, and WNT10A were elevated in tumor specimens derived from lincROR overexpression group (Fig 5E). These findings suggested that lincROR overexpression activated Wnt/β-catenin signaling and promoted CRC tumorigenesis *in vivo*.

## Discussion

LincROR, widely recognized as an oncogene, has been reported to exhibit abnormally high expression and play crucial roles in various tumors [8]. For instance, lincROR is upregulated in gastric carcinoma and promotes cancer cell proliferation and metastasis [9]. Furthermore, lincROR is involved in the resistance to doxorubicin in hepatocellular carcinoma [10] and tumor growth in esophageal squamous cell carcinoma [11]. Recent studies indicated that lincROR enhanced cell proliferation [12] and decreased sensitivity to radiotherapy in CRC [13]. LincROR was also shown to be involved in the anti-tumour activity of berberine in CRC in our previous research [14]. However, the exact roles and mechanism of the lincROR in CRC remain unclear. In the current study, we demonstrated that lincROR was upregulated in most CRC cell lines and mediated CRC cell proliferation and tumor growth in *vitro* and *in vivo*. These findings indicate that lincROR elevation may be a common phenomenon in CRC development, and it can serve as a proto-oncogene in CRC tumorigenesis.

Multiple cellular signalling pathways such as Wnt/β-catenin, AKT/PI3K [15], TGF-β [16], MAPK/ERK [7] have been reported to be implicated in the lincROR-mediated tumorigenesis. Of which, the Wnt/β-catenin pathway is particularly significant in CRC development. Over 94% CRC patients exhibit mutations in one or more members of the Wnt signalling family, with APC and β-catenin being the most frequently affected (in about 80% of cases) [17]. However, whether this signalling participates in lincROR-mediated CRC tumorigenesis remains unclear. In the present study, our results indicated that lincROR knockdown induced the inactivation of Wnt/β-catenin signalling *via* examining the luciferase activity and the expression of β-catenin and several downstream target genes of this signalling.

A great deal of evidence has demonstrated that lncRNAs function as ceRNAs by binding to miRNAs, thus subsequently antagonize their functions and led to the de-repression of their endogenous targets [18]. The lncRNA-miRNA-mRNA regulatory network is theorized to play an indispensable role in various tumors. Based on the ceRNA hypothesis, miR-145 was predicted as a potential target of lincROR in CRC cells. The further dual-luciferase reporter assays confirmed that lincROR directly bound to miR-145 in CRC cells. This result was consistent with previous reports on endometrial cancer stem cells [19], triple-negative breast cancer cells, and pancreatic cancer cells [20]. As a well-known tumor suppressor, miR-145 has been demonstrated to be down-regulated in various tumors and inhibited the malignant processes by inhibiting the expression of target genes [21]. MiR-145 has been reported to suppress the proliferation, cell cycle, apoptosis, angiogenesis, and migration of CRC cells through directly targeting several oncogenes such as c-Myc [22], CDK1 [23], and Friend leukemia virus integration 1 gene (FLI1) [24]. In this study, WNT2B and WNT10A were identified as two potential target candidates of miR-145, and further dual luciferase activity and qRT-PCR examination confirmed that miR-145 directly targeted WNT2B and WNT10A in CRC cells. WNT2B and WNT10A belong to the canonical Wnt family members and closely associated with the activation of Wnt/β-catenin signalling [25,26]. As expected, our results demonstrated that miR-145 suppressed the Wnt/β-catenin signalling by specifically inhibiting the expression of WNT2B and WNT10A in CRC cells. Furthermore, enforced expression of lincROR successfully rescued the inhibition of Wnt/β-catenin signalling induced by miR-145. These findings suggested that lincROR facilitated CRC cell growth *via* the miR-145/WNT2B/WNT10A/Wnt/β-catenin signalling regulatory axis.

LncRNA has been considered to be a potential therapeutic target or diagnostic marker for cancer treatment [27–29]. Our findings indicated that lincROR was abnormally upregulated in CRC and promoted tumorigenesis by activating miR-145/WNT2B/WNT10A/Wnt/β-catenin signalling regulatory axis. Therefore, LincROR has the potential to be a therapeutic target for colorectal cancer. However, the clinical significance of lincROR could not be in-depth explored in the present study due to a lack of clinical CRC samples or patient-derived models (xenografts or organoids). Based on the preceding reference [30], we can utilize the proteotranscriptomic analysis by patient-derived organoids after knockdown or overexpression of lincROR to further demonstrate the importance of lincROR/WNT2B/WNT10A/WNT/β-catenin regulatory axis in CRC in the future. Moreover, the gene ROR was reported to be associated with the development of TGF-β induced tumors [31], we justly wondered whether this lincROR could act as a therapeutic target for TGF-β related colorectal cancer. Therefore, more clinical and basic studies will be conducted in our future research plan.

Notably, the precise classification of CRC based on consensus molecular subtype (CMS) may provide more advantageous for the therapeutic and prognosis of CRC patients [32]. Our results identified that lincROR exerted a significant effect on tumorigenesis through activating the WNT/β-catenin signalling, which made us to speculate a close association between lincROR expression and the CRC patients with abnormally high WNT signalling activation, namely CMS2 patients. We therefore suggest that lincROR may be a potential diagnostic marker and therapeutic target for CMS2 CRC patients.

In summary, our results have demonstrated the oncogenic role of lincROR in CRC *via* serving as ceRNA and stimulating Wnt/β-catenin signalling. This finding provides strong support for the development of effective molecular targets for CRC patients.

## Material and methods

### Cell culture

The human colorectal cancer cell lines including HCT116, HT29, HCT15, HCT8, DLD1, RKO, and COLO201 cells, as well as Fetal human colon (FHC) cells, were cultured in 1640 medium (RPMI-1640, Sigma-Aldrich Corp, St. Louis, MO, USA) supplemented with 10% fetal bovine serum (FBS, Thermo Fisher Scientific, Green Island, NY, USA) and 1% Penicillin and Streptomycin (P/S, Thermo Fisher Scientific). The HEK293T cells (American Type Culture Collection, Manassas, VA, USA) were cultured in DMEM (Sigma-Aldrich Corp) with 10% FBS and 1% P/S. All cells were cultured at 37°C in humidified cell incubator with 5% CO_2_.

### Cell transfection

Hsa-miR-145/NC and anti-hsa-miR-145/anti-NC were purchased from GenePharma (Shanghai, China), and their sequences were shown in S1 Table. These were transfected into cells by Lipofectamine 3000 (Invitrogen, USA) following the instructions of the manufacturer.

### Cell viability assays

SW620 cells were seeded into 96-well plates at a density of 5×10^3^ cells/well. Subsequently, the Cell Counting Assay Kit-8 solution (Beyotime, Shanghai China) was incorporated and the mixture was incubated for an additional hour at 37°C. The absorbance measurements were measured at 450 nm utilizing a Hybrid Multi-Mode Microplate Reader (Tecan, Switzerland). All the experiments were performed in triplicates.

### Colony formation assays

Cells were seed in 6-well plates and maintained for 14 days. The colonies were stained with Giemsa (Beyotime, Shanghai, China), and the images of the colony formation were taken by the ImmunoSpot analyzers (CTL, USA). The number of colonies was quantified by Immuno-Spot® Version 6.0 Academic system (CTL, USA).

### Flow cytometry examination

For apoptosis analysis, cells were collected after transfected with miRNA oligos for 48 h, and followed by annexin V and propidium iodide (PI) incubation according to the protocol of the Apoptosis Detection Kit (KeyGEN, Nanjing, China). For cell cycle analysis, cells were harvested and stained with the dye from the Cell Cycle Detection Kit (KeyGEN) following the manufacturer’s protocol.

### Plasmid construction

The construction of lincROR overexpression (plincROR) and knockdown (shROR) has been described previously [33]. For luciferase reporter construction, fragments of lincROR, WNT2B, and WNT10A contained the predicted miR-145 binding site, as well as their corresponding site-mutated sequence were amplified. These fragments were then respectively cloned into a pmir-GLO vector (Promega, Madison, WI, USA) to generate WT and MUT luciferase reporter vector. The primers of the dual-luciferase reporter constructions were displayed in S2 Table.

### Dual-luciferase assays

HEK293T cells were co-transfected with the luciferase reporters and miRNA oligos by lipofectamine 3000. After 48 hours, the luciferase activities were detected using a dual-luciferase reporter system (Cat E1910, Promega, USA). Renilla luciferase activity served as a control to normalize the firefly luciferase activity.

### RNA Extraction, Reverse Transcription, and Quantitative Real-time Polymerase Chain Reaction (qRT-PCR)

The total RNA was extracted by Animal Total RNA Isolation Kit (Cat RE-03014, FOREGENE, Chengdu, China) and then reverse transcribed to cDNA using Prime Script RT Reagent Kit (Cat RR036A, Takara, Japan). The real-time polymerase chain reaction was performed using Power up SYBR Green Master Mix (Cat A25742, Thermo Fisher Scientific, Waltham, MA) on an applied Light-Cycler480 System (Roche, Basel, Switzerland). GAPDH or U6 was used as the endogenous controls. All the calculations were analyzed for each gene by the 2^–ΔΔCt^ method. The primers sequences used in this study are listed in S3 Table.

### Western blot

The cells were lysed using RIPA lysis buffer (Cat P0013B, Beyotime), and the supernatant fraction was collected by centrifugation at 13000 g for 15 minutes. The protein concentration was quantified using a BCA assay. Equal amounts of protein were separated by 10% SDS-PAGE, transferred to a PVDF membrane (Cat. NO. SLGV004SL-1, Millipore, MA, USA). The membrane was blocked with 5% skimmed milk for 1 h at room temperature. The following antibodies were used to probe membrane at 4°C overnight: β-catenin (1:2000; Cat 8084S, Cell Signalling Technology, USA), WNT2B (1:2000; Cat ab178418, Abcam, UK), WNT10A (1:1000; Cat NBP1-76916, Novus Biologicals, USA), and GAPDH (1:2000; Cat 5174S, Cell Signalling Technology, USA). The membranes were further incubated with corresponding HRP-conjugated secondary antibody. Finally, the protein bands were visualized using a chemiluminescence system (ECL, Cat WBKLS0500, Merck Millipore, Germany).

### Xenografts mouse model

The animals were purchased from Laboratory Animal Services Centre of Southern Medical University, and this experiment was approved by the Southern Medical University Institutional Animal Care and Use Committee (IACUC). Prior to conducting animal experiments, we all completed the animal experiment training at IACUC. Ten nude BALB/c mice were randomly divided into two equal groups, with each mouse in the group subcutaneously injected into the dorsal flank with stably linc-ROR overexpressing SW620 cells (1.5×10^6^ cells per mouse) (n = 5 mice/group). After one week, tumors were measured every three days. The tumor volumes were calculated according to the formula: V = length × width^2^/2. Approximately 28 days later, when the tumor volume in mice reached 1000 cubic millimeters, all mice were sacrificed by cervical dislocation within one minute under anesthesia with 3% isoflurane and tumors were dissected for further examination. No animals died before reaching the criteria for euthanasia.

### Immunohistochemistry and immunofluorescence analysis

The tumor tissues were embedded into the paraffin and sectioned, followed by immunohistochemistry and immunofluorescence staining using standardized protocols. Briefly, paraffin sections underwent de-waxing, hydrated, and treated with citric acid buffer (0.01M, PH = 6.0) for antigen retrieval. Subsequently, the tumor sections were blocked with goat serum (Cat AR0009, Boster Biological Technology Co. Ltd, USA) and incubated overnight at 4°C with primary antibodies: β-catenin (1:3000, PTG, China) or Ki-67 (Cat 9129S, 1:100, Abcam, UK). Next, they were incubated for 1 h in the dark with secondary antibody: either goat anti-rabbit HRP (1:2000, Cat ab205718, Abcam) or Donkey anti-rabbit, Alexa Fluor 594 (Cat A21207, Life, Invitrogen). Visualization was achieved by using 3, 3’-diaminobenzidine substrate (Cat C1005, Beyotime, Shanghai, China), followed by counterstaining with hematoxylin or DAPI (Cat C1005, Beyotime, Shanghai, China). Representative images were captured using a Zeiss microscope (Zeiss, Germany).

### Statistical analysis

Experimental data are expressed as the means ± SD. Graph Pad Prism 8.0.2 software was used for all statistical analysis. The Brown-Forsythe test is utilized to evaluate the variance homogeneity among the groups undergoing statistical comparison. The observed variance was found to be similar between the groups under consideration for statistical comparison. For comparing differences between two groups, an unpaired t-test was employed. In cases where the data comprised three or more groups, a one-way Analysis of Variance (ANOVA) coupled with Tukey’s multiple comparison test was utilized to discern the impact of a single independent variable. *P* values<0.05 was considered to be a statistical significance.

## Supporting information

S1 Fig MiR-145 induced the cell cycle arrest and apoptosis in SW620 cells. **(A)** The cell cycle was examined in SW620 cells with miR-145 transfection. **(B)** The apoptotic cells were tested in SW620 cells with anti-miR-145 transfection. Data were shown as mean ± SD (n = 3). *, *P* < 0.05; ***, *P* < 0.001; versus the corresponding control group.
(DOCX)

S1 Table The sequences of miRNA. (DOCX)

S2 Table Primer sequences for plasmid construction. (DOCX)

S3 Table Primer sequences for qPCR examination. (DOCX)

S1 Raw images Raw blot images. (PPTX)

## References

[1] Sung H, Ferlay J, Siegel RL, Laversanne M, Soerjomataram I, Jemal A, et al. Global Cancer Statistics 2020: GLOBOCAN Estimates of Incidence and Mortality Worldwide for 36 Cancers in 185 Countries. CA Cancer J Clin. 2021;71(3):209–49. Epub 2021/02/05. doi: 10.3322/caac.21660 . 33538338

[2] Pauli A, Rinn JL, Schier AF. Non-coding RNAs as regulators of embryogenesis. Nat Rev Genet. 2011;12(2):136–49. Epub 2011/01/20. doi: 10.1038/nrg2904 ; PubMed Central PMCID: PMC4081495. 21245830 PMC4081495

[3] Weidle UH, Birzele F, Kollmorgen G, Ruger R. Long Non-coding RNAs and their Role in Metastasis. Cancer Genomics Proteomics. 2017;14(3):143–60. Epub 2017/04/28. doi: 10.21873/cgp.20027 ; PubMed Central PMCID: PMC5420816. 28446530 PMC5420816

[4] Mi Y, Li Y, He Z, Chen D, Hong Q, You J. Upregulation of Linc-ROR Promotes the Proliferation, Migration, and Invasion of Gastric Cancer Cells Through miR-212-3p/FGF7 Axis. Cancer Manag Res. 2021;13:899–912. Epub 2021/02/11. doi: 10.2147/CMAR.S287775 ; PubMed Central PMCID: PMC7867499. 33564265 PMC7867499

[5] Shao J, Shi CJ, Li Y, Zhang FW, Pan FF, Fu WM, et al. LincROR Mediates the Suppressive Effects of Curcumin on Hepatocellular Carcinoma Through Inactivating Wnt/beta-Catenin Signaling. Front Pharmacol. 2020;11:847. Epub 2020/07/28. doi: 10.3389/fphar.2020.00847 ; PubMed Central PMCID: PMC7351502. 32714183 PMC7351502

[6] Pena-Flores JA, Enriquez-Espinoza D, Muela-Campos D, Alvarez-Ramirez A, Saenz A, Barraza-Gomez AA, et al. Functional Relevance of the Long Intergenic Non-Coding RNA Regulator of Reprogramming (Linc-ROR) in Cancer Proliferation, Metastasis, and Drug Resistance. Noncoding RNA. 2023;9(1). Epub 2023/02/25. doi: 10.3390/ncrna9010012 ; PubMed Central PMCID: PMC9965135. 36827545 PMC9965135

[7] Feng L, Shi L, Lu YF, Wang B, Tang T, Fu WM, et al. Linc-ROR Promotes Osteogenic Differentiation of Mesenchymal Stem Cells by Functioning as a Competing Endogenous RNA for miR-138 and miR-145. Mol Ther Nucleic Acids. 2018;11:345–53. Epub 2018/06/03. doi: 10.1016/j.omtn.2018.03.004 ; PubMed Central PMCID: PMC5992460. 29858070 PMC5992460

[8] Chen W, Yang J, Fang H, Li L, Sun J. Relevance Function of Linc-ROR in the Pathogenesis of Cancer. Front Cell Dev Biol. 2020;8:696. Epub 2020/08/28. doi: 10.3389/fcell.2020.00696 ; PubMed Central PMCID: PMC7432147. 32850817 PMC7432147

[9] Bai L, Zhuang Y, Xie J, Liu K, Yin S, Yan F. SOX2-Induced Linc-ROR Upregulation Inhibits Gastric Carcinoma Cell Proliferation and Metastasis Via the miR-580-3p/ANXA10 Pathway. Biochem Genet. 2023;61(3):1113–27. Epub 2022/12/01. doi: 10.1007/s10528-022-10300-w . 36451051

[10] Zhang Y, Wu W, Sun Q, Ye L, Zhou D, Wang W. linc‑ROR facilitates hepatocellular carcinoma resistance to doxorubicin by regulating TWIST1‑mediated epithelial‑mesenchymal transition. Mol Med Rep. 2021;23(5). Epub 2021/03/25. doi: 10.3892/mmr.2021.11979 ; PubMed Central PMCID: PMC7974311. 33760121 PMC7974311

[11] Gao H, Wang T, Zhang P, Shang M, Gao Z, Yang F, et al. Linc-ROR regulates apoptosis in esophageal squamous cell carcinoma via modulation of p53 ubiquitination by targeting miR-204-5p/MDM2. J Cell Physiol. 2020;235(3):2325–35. Epub 2019/09/22. doi: 10.1002/jcp.29139 . 31541467

[12] Li X, Chen W, Jia J, You Z, Hu C, Zhuang Y, et al. The Long Non-Coding RNA-RoR Promotes the Tumorigenesis of Human Colorectal Cancer by Targeting miR-6833-3p Through SMC4. Onco Targets Ther. 2020;13:2573–81. Epub 2020/04/11. doi: 10.2147/OTT.S238947 ; PubMed Central PMCID: PMC7109305. 32273727 PMC7109305

[13] Yang P, Yang Y, An W, Xu J, Zhang G, Jie J, et al. The long noncoding RNA-ROR promotes the resistance of radiotherapy for human colorectal cancer cells by targeting the p53/miR-145 pathway. J Gastroenterol Hepatol. 2017;32(4):837–45. Epub 2016/10/04. doi: 10.1111/jgh.13606 . 27696511

[14] Li SY, Shi CJ, Fu WM, Zhang JF. Berberine inhibits tumour growth in vivo and in vitro through suppressing the lincROR-Wnt/beta-catenin regulatory axis in colorectal cancer. J Pharm Pharmacol. 2023;75(1):129–38. Epub 2022/09/22. doi: 10.1093/jpp/rgac067 . 36130331

[15] Ma X, Zhang H, Li Q, Schiferle E, Qin Y, Xiao S, et al. FOXM1 Promotes Head and Neck Squamous Cell Carcinoma via Activation of the Linc-ROR/LMO4/AKT/PI3K Axis. Front Oncol. 2021;11:658712. Epub 2021/08/28. doi: 10.3389/fonc.2021.658712 ; PubMed Central PMCID: PMC8383294. 34447693 PMC8383294

[16] Hou L, Tu J, Cheng F, Yang H, Yu F, Wang M, et al. Long noncoding RNA ROR promotes breast cancer by regulating the TGF-beta pathway. Cancer Cell Int. 2018;18:142. Epub 2018/09/27. doi: 10.1186/s12935-018-0638-4 ; PubMed Central PMCID: PMC6145201. 30250400 PMC6145201

[17] Zhang L, Shay JW. Multiple Roles of APC and its Therapeutic Implications in Colorectal Cancer. J Natl Cancer Inst. 2017;109(8). Epub 2017/04/20. doi: 10.1093/jnci/djw332 ; PubMed Central PMCID: PMC5963831. 28423402 PMC5963831

[18] Tay Y, Rinn J, Pandolfi PP. The multilayered complexity of ceRNA crosstalk and competition. Nature. 2014;505(7483):344–52. Epub 2014/01/17. doi: 10.1038/nature12986 ; PubMed Central PMCID: PMC4113481. 24429633 PMC4113481

[19] Zhou X, Gao Q, Wang J, Zhang X, Liu K, Duan Z. Linc-RNA-RoR acts as a "sponge" against mediation of the differentiation of endometrial cancer stem cells by microRNA-145. Gynecol Oncol. 2014;133(2):333–9. Epub 2014/03/05. doi: 10.1016/j.ygyno.2014.02.033 . 24589415

[20] Eades G, Wolfson B, Zhang Y, Li Q, Yao Y, Zhou Q. lincRNA-RoR and miR-145 regulate invasion in triple-negative breast cancer via targeting ARF6. Mol Cancer Res. 2015;13(2):330–8. Epub 2014/09/26. doi: 10.1158/1541-7786.MCR-14-0251 ; PubMed Central PMCID: PMC4336811. 25253741 PMC4336811

[21] Zeinali T, Mansoori B, Mohammadi A, Baradaran B. Regulatory mechanisms of miR-145 expression and the importance of its function in cancer metastasis. Biomed Pharmacother. 2019;109:195–207. Epub 2018/11/06. doi: 10.1016/j.biopha.2018.10.037 . 30396077

[22] Li J, Zhang S, Zou Y, Wu L, Pei M, Jiang Y. miR-145 promotes miR-133b expression through c-myc and DNMT3A-mediated methylation in ovarian cancer cells. J Cell Physiol. 2020;235(5):4291–301. Epub 2019/10/16. doi: 10.1002/jcp.29306 . 31612498

[23] Zhou L, Mu D, Chen Y. LINC00958 Targets miR-145-3p/CDK1 Axis to Aggravate the Malignancy of Colon Cancer. Ann Clin Lab Sci. 2022;52(5):695–706. Epub 2022/10/20. . 36261176

[24] Chen Z, Xu W, Zhang D, Chu J, Shen S, Ma Y, et al. circCAMSAP1 promotes osteosarcoma progression and metastasis by sponging miR-145-5p and regulating FLI1 expression. Mol Ther Nucleic Acids. 2021;23:1120–35. Epub 2021/03/06. doi: 10.1016/j.omtn.2020.12.013 ; PubMed Central PMCID: PMC7901030. 33664993 PMC7901030

[25] Katoh M, Hirai M, Sugimura T, Terada M. Cloning, expression and chromosomal localization of Wnt-13, a novel member of the Wnt gene family. Oncogene. 1996;13(4):873–6. Epub 1996/08/15. . 8761309

[26] Kirikoshi H, Sekihara H, Katoh M. Molecular cloning and characterization of human WNT11. Int J Mol Med. 2001;8(6):651–6. Epub 2001/11/17. doi: 10.3892/ijmm.8.6.651 . 11712081

[27] Liu C, Shen A, Song J, Cheng L, Zhang M, Wang Y, et al. LncRNA-CCAT5-mediated crosstalk between Wnt/beta-Catenin and STAT3 signaling suggests novel therapeutic approaches for metastatic gastric cancer with high Wnt activity. Cancer Commun (Lond). 2024;44(1):76–100. Epub 2023/11/27. doi: 10.1002/cac2.12507 ; PubMed Central PMCID: PMC10794011. 38010289 PMC10794011

[28] Zhou Y, Shao Y, Hu W, Zhang J, Shi Y, Kong X, et al. A novel long noncoding RNA SP100-AS1 induces radioresistance of colorectal cancer via sponging miR-622 and stabilizing ATG3. Cell Death Differ. 2023;30(1):111–24. Epub 2022/08/18. doi: 10.1038/s41418-022-01049-1 ; PubMed Central PMCID: PMC9883267. 35978049 PMC9883267

[29] Coan M, Haefliger S, Ounzain S, Johnson R. Targeting and engineering long non-coding RNAs for cancer therapy. Nat Rev Genet. 2024;25(8):578–95. Epub 2024/03/01. doi: 10.1038/s41576-024-00693-2 . 38424237

[30] Papaccio F, Garcia-Mico B, Gimeno-Valiente F, Cabeza-Segura M, Gambardella V, Gutierrez-Bravo MF, et al. "Proteotranscriptomic analysis of advanced colorectal cancer patient derived organoids for drug sensitivity prediction". J Exp Clin Cancer Res. 2023;42(1):8. Epub 2023/01/06. doi: 10.1186/s13046-022-02591-z ; PubMed Central PMCID: PMC9817273. 36604765 PMC9817273

[31] Tarazona N, Gimeno-Valiente F, Gambardella V, Huerta M, Rosello S, Zuniga S, et al. Detection of postoperative plasma circulating tumour DNA and lack of CDX2 expression as markers of recurrence in patients with localised colon cancer. ESMO Open. 2020;5(5):e000847. Epub 2020/09/25. doi: 10.1136/esmoopen-2020-000847 ; PubMed Central PMCID: PMC7513635. 32967918 PMC7513635

[32] Singh MP, Rai S, Pandey A, Singh NK, Srivastava S. Molecular subtypes of colorectal cancer: An emerging therapeutic opportunity for personalized medicine. Genes Dis. 2021;8(2):133–45. Epub 2019/10/30. doi: 10.1016/j.gendis.2019.10.013 ; PubMed Central PMCID: PMC8099693. 33997160 PMC8099693

[33] Huang J, Zhang A, Ho TT, Zhang Z, Zhou N, Ding X, et al. Linc-RoR promotes c-Myc expression through hnRNP I and AUF1. Nucleic Acids Res. 2016;44(7):3059–69. Epub 2015/12/15. doi: 10.1093/nar/gkv1353 ; PubMed Central PMCID: PMC4838338. 26656491 PMC4838338

