# Supplementary material for: LincROR promotes tumor growth of colorectal cancer through the miR-145/WNT2B/WNT10A/Wnt/β-catenin regulatory axis
Source: PLoS One. 2024 Nov 15;19(11):e0312417. doi: 10.1371/journal.pone.0312417 (PMC11567539; doi:10.1371/journal.pone.0312417)
Supplement: S1 Fig — (A) The cell cycle was examined in SW620 cells with miR-145 transfection. (B) The apoptotic cells were tested in SW620 cells with anti-miR-145 transfection. Data were shown as mean ± SD (n = 3). *, P < 0.05; ***, P < 0.001; versus the corresponding control group. (DOCX) [file pone.0312417.s001.docx]

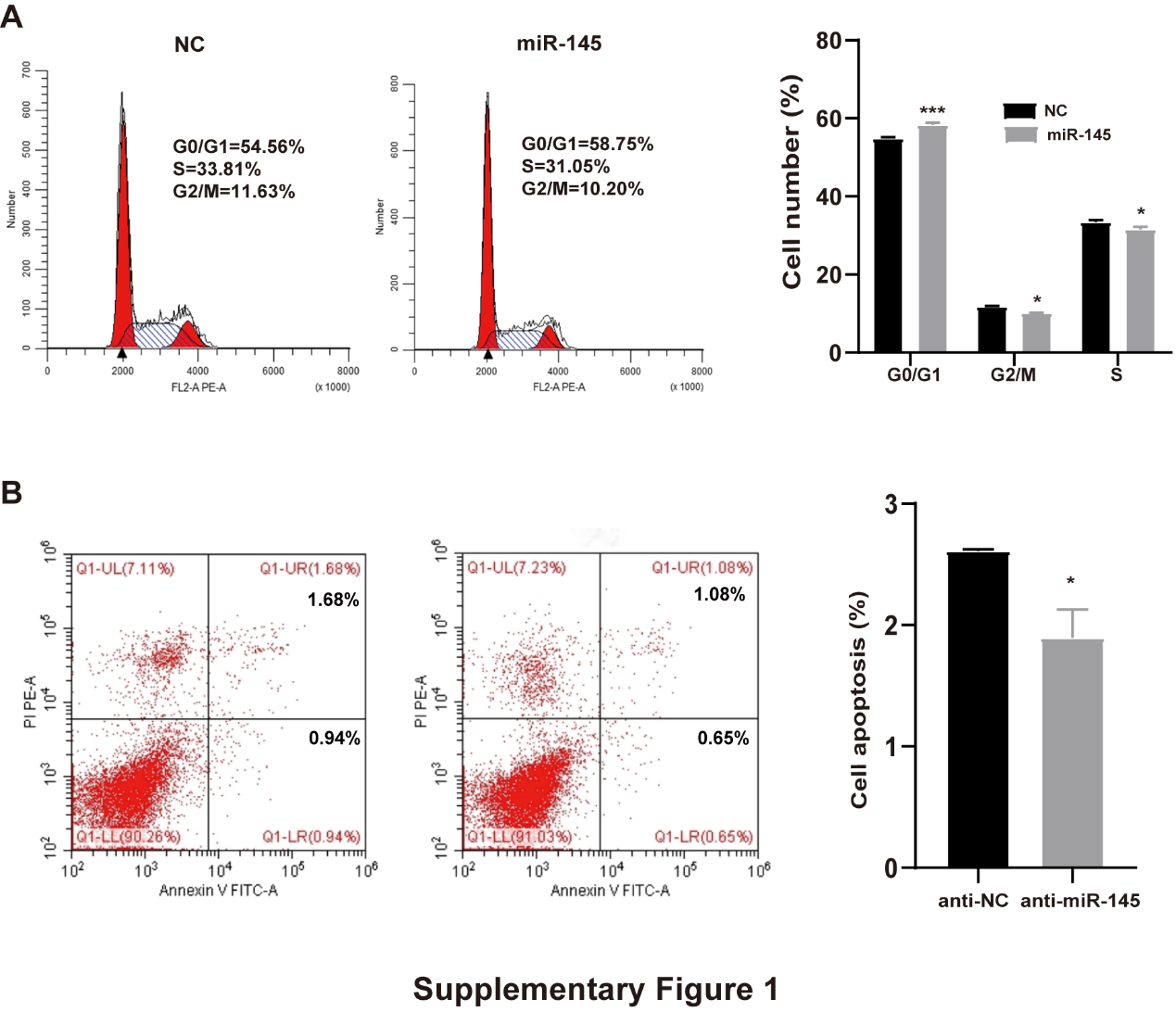
 **S1 Fig. MiR-145 induced the cell cycle arrest and apoptosis in SW620 cells. (A)** The cell cycle was examined in SW620 cells with miR-145 transfection. **(B)** The apoptotic cells were tested in SW620 cells with anti-miR-145 transfection. Data were shown as mean ± SD (n = 3). *, *P* < 0.05; ***, *P* < 0.001 ; versus the corresponding control group.
