# Supplementary material for: LincROR promotes tumor growth of colorectal cancer through the miR-145/WNT2B/WNT10A/Wnt/β-catenin regulatory axis
Source: PLoS One. 2024 Nov 15;19(11):e0312417. doi: 10.1371/journal.pone.0312417 (PMC11567539; doi:10.1371/journal.pone.0312417)
Supplement: S1 Table — (DOCX) [file pone.0312417.s002.docx]

**Supplementary Table 1.** The sequences of miRNA.

| **Name** | **siRNA, mimics, and inhibitors (5’-3’)** |
| --- | --- |
| **has-miR-145 mimics** | GUCCAGUUUUCCCAGGAAUCCCU |
| **mimics negative control** | UUCUCCGAACGUGUCACGUTT |
| **anti-has-miR-145** | AGGGAUUCCUGGGAAAACUGGAC |
| **anti-NC** | CAGUACUUUUGUGUAGUACAA |
