# Supplementary material for: LincROR promotes tumor growth of colorectal cancer through the miR-145/WNT2B/WNT10A/Wnt/β-catenin regulatory axis
Source: PLoS One. 2024 Nov 15;19(11):e0312417. doi: 10.1371/journal.pone.0312417 (PMC11567539; doi:10.1371/journal.pone.0312417)
Supplement: S2 Table — (DOCX) [file pone.0312417.s003.docx]

**Supplementary Table 2.** Primer sequences for plasmid construction.

| \| **Name** \| **Primer sequences used for plasmid construction (5’-3’)** \| \| --- \| --- \| | |
| --- | --- | --- | --- |
| **LincROR-WT-F** | CTAGCTAGCCCTTGCACACCCTGATAACCT |
| **LincROR-WT-R** | CTAGTCTAGACTGAAGTCTCTTCTTTGGGGC |
| **WNT2B-WT -F** | CTAGCTAGCAGAATGGATCCGAGAGTGTCAGC |
| **WNT2B-WT -R** | CTAGTCTAGA AATCTGAGAGTGCACGCCAG |
| **WNT10A-WT -F** | CTAGCTAGCGTGCTCAATGCCAACACAGT |
| **WNT10A-WT -R** | CTAGTCTAGATTACCACGCTGCAGTGCAT |
| **WNT2B-MUT -F** | CTAGCTAGCACCACACCGTCTTTGGCCGT |
| **WNT2B-MUT -R** | CTAGTCTAGAAGTGCACGCCAGCAGGTGC |
| **WNT10A-MUT -F** | CTAGCTAGCGTGCTCAATGCCAACACAGTG |
| **WNT10A-MUT -R** | ACGCGTCGACTTGTAAGCGGTGCAGCTTCC |
