# Supplementary material for: LincROR promotes tumor growth of colorectal cancer through the miR-145/WNT2B/WNT10A/Wnt/β-catenin regulatory axis
Source: PLoS One. 2024 Nov 15;19(11):e0312417. doi: 10.1371/journal.pone.0312417 (PMC11567539; doi:10.1371/journal.pone.0312417)
Supplement: S3 Table — (DOCX) [file pone.0312417.s004.docx]

**Supplementary Table 3.** Primer sequences for qPCR examination.

| \| **Name** \| **Primer sequences used for plasmid construction (5’-3’)** \| \| --- \| --- \| | |
| --- | --- | --- | --- |
| **Axin2-F** | CAACACCAGGCGGAACGAA |
| **Axin2-R** | GCCCAATAAGGAGTGTAAGGACT |
| **β-catenin-F** | CATCTACACAGTTTGATGCTGCT |
| **β-catenin-R** | GCAGTTTTGTCAGTTCAGGGA |
| **CD44-F** | CTGCCGCTTTGCAGGTGTA |
| **CD44-R** | CATTGTGGGCAAGGTGCTATT |
| **Oct3/4-F** | CTGGGTTGATCCTCGGACCT |
| **Oct3/4-R** | CCATCGGAGTTGCTCTCCA |
| **Survivin-F** | AGGACCACCGCATCTCTACAT |
| **Survivin-R** | AAGTCTGGCTCGTTCTCAGTG |
| **WNT2B-F** | GGGGCACGAGTGATCTGTG |
| **WNT2B-R** | GCATGATGTCTGGGTAACGCT |
| **WNT10A-F** | GGTCAGCACCCAATGACATTC |
| **WNT10A-R** | TGGATGGCGATCTGGATGC |
| **LincROR-F** | CTGGCTTTCTGGTTTGACG |
| **LincROR-R** | CAGGAGGTTACTGGACTTGGAG |
| **GAPDH-F** | GGAGCGAGATCCCTCCAAAAT |
| **GAPDH-R** | GGCTGTTGTCATACTTCTCATGG |
