# Supplementary material for: LincROR promotes tumor growth of colorectal cancer through the miR-145/WNT2B/WNT10A/Wnt/β-catenin regulatory axis
Source: PLoS One. 2024 Nov 15;19(11):e0312417. doi: 10.1371/journal.pone.0312417 (PMC11567539; doi:10.1371/journal.pone.0312417)
Supplement: S1 Raw images — (PPTX) [file pone.0312417.s005.pptx]

## Slide 1
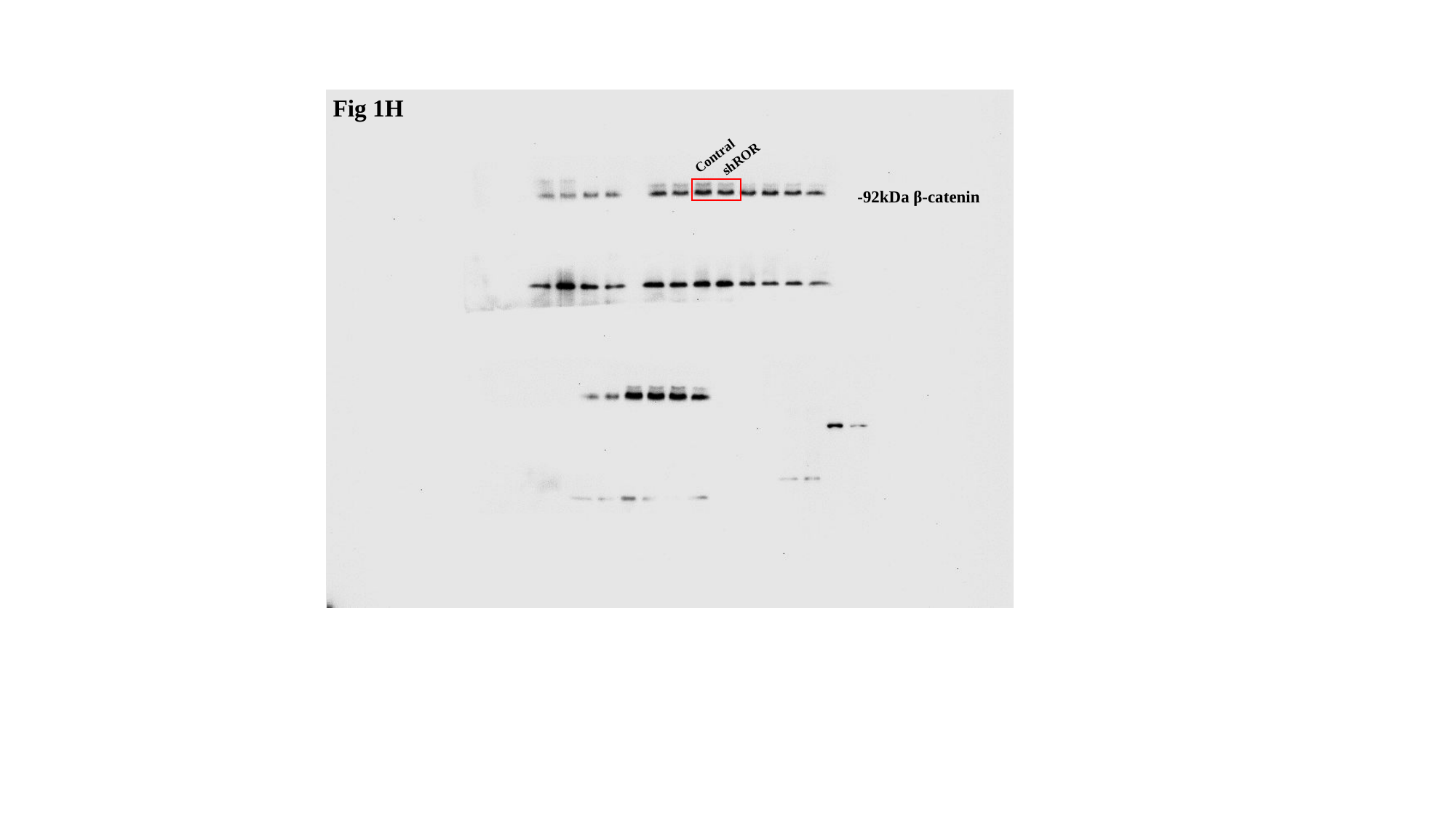

Fig 1H
Contral
shROR
-92kDa β-catenin

## Slide 2
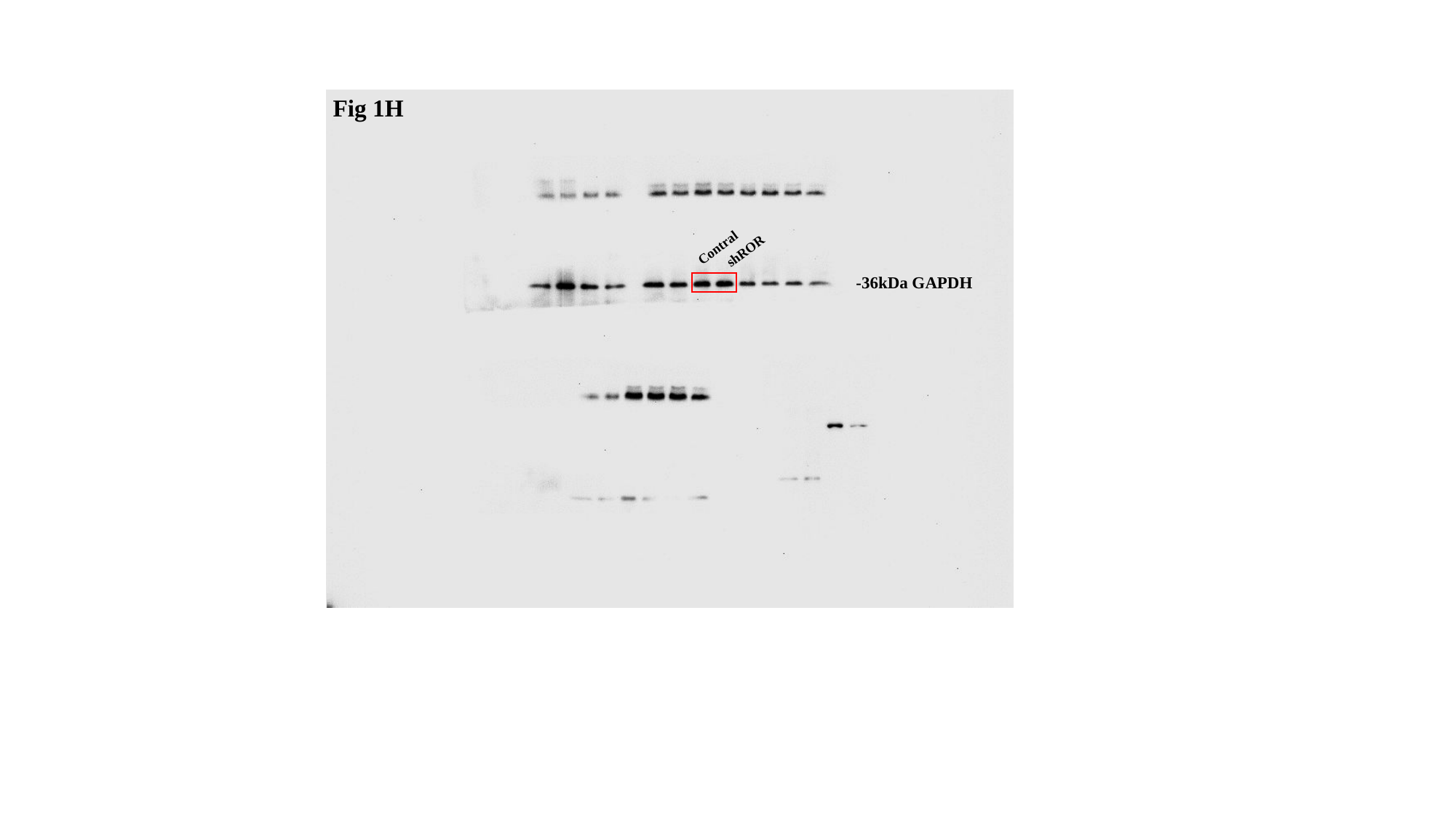

Fig 1H
Contral
shROR
-36kDa GAPDH

## Slide 3
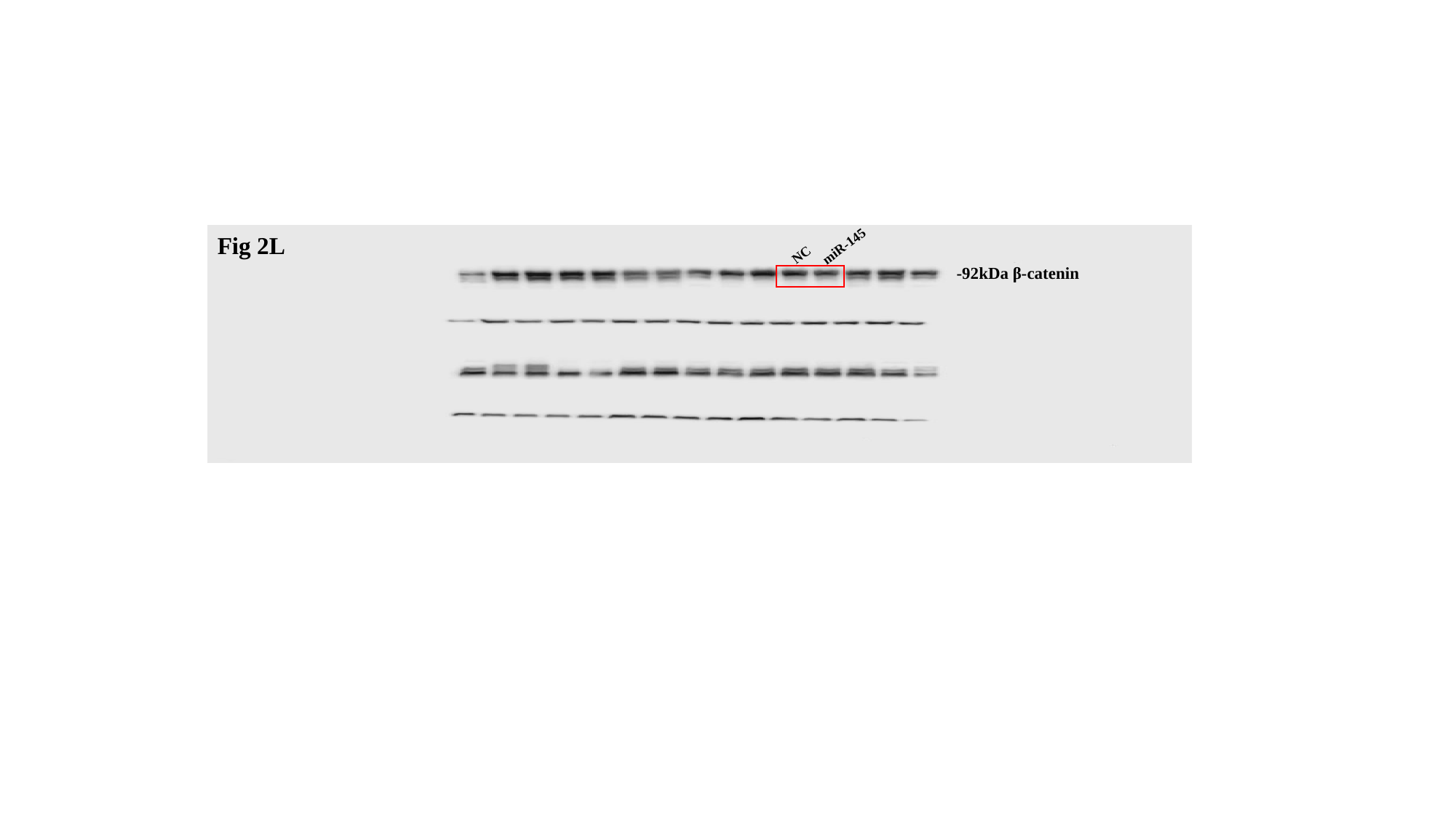

Fig 2L
miR-145
NC
-92kDa β-catenin

## Slide 4
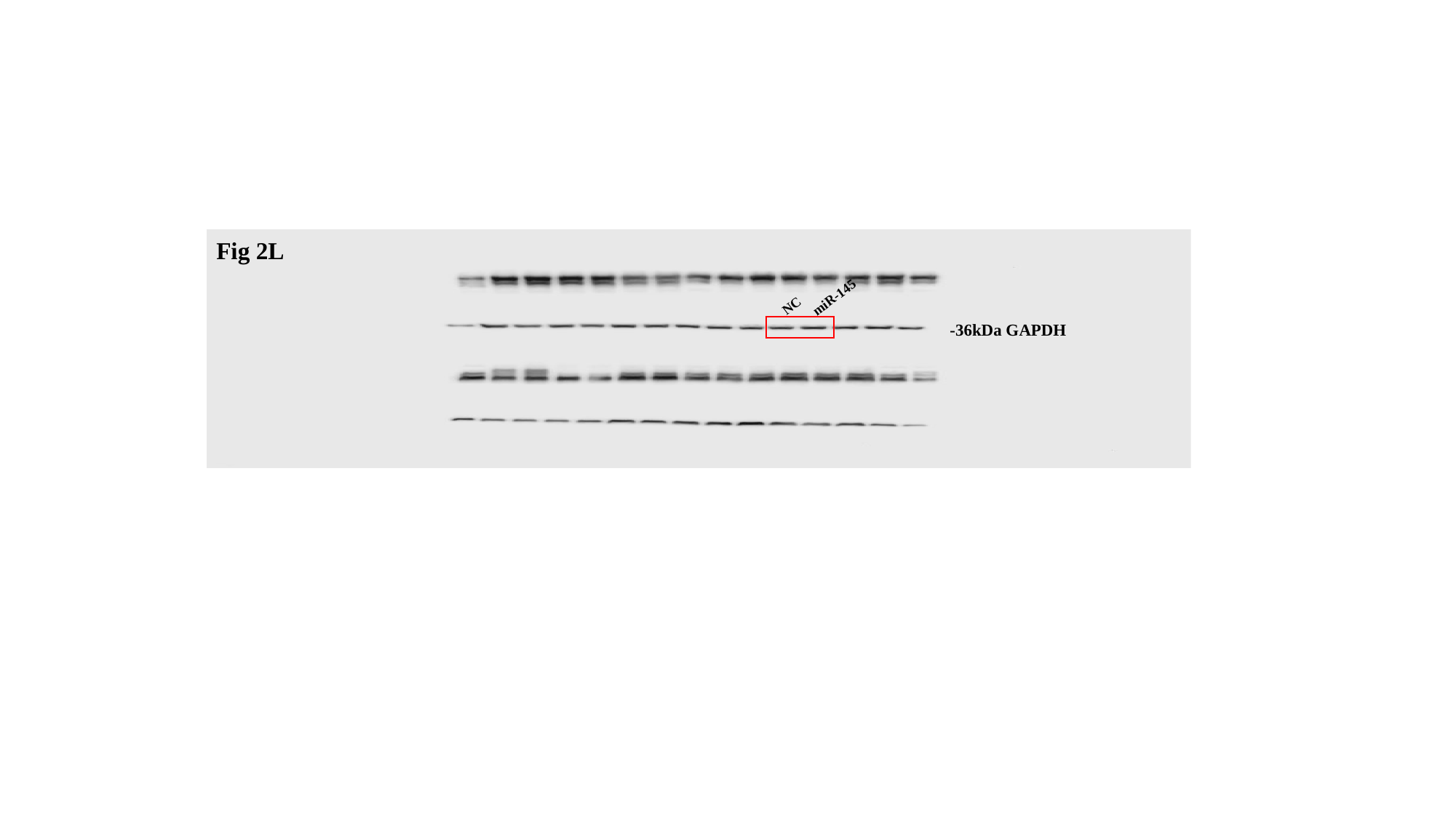

Fig 2L
miR-145
NC
-36kDa GAPDH

## Slide 5
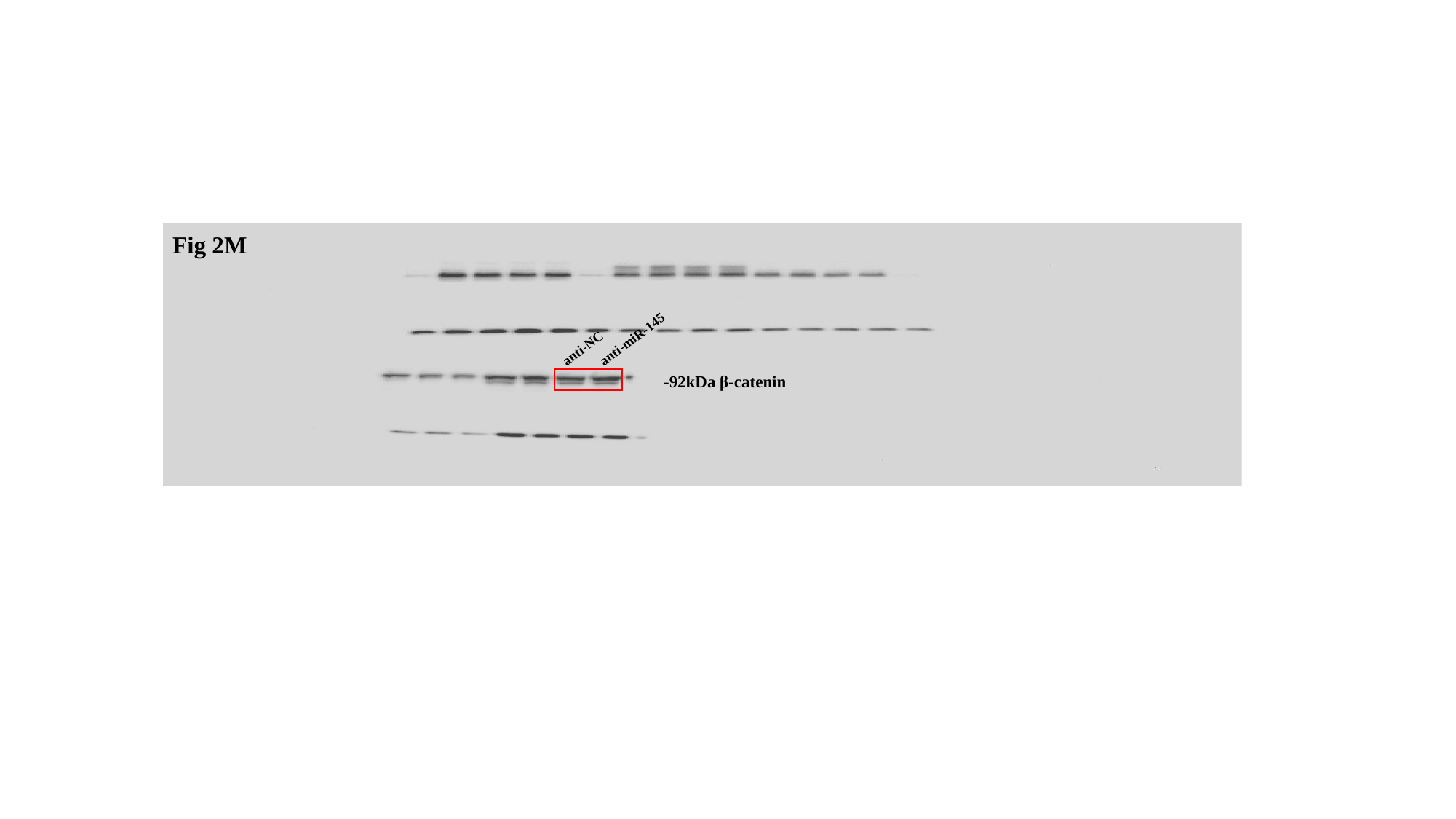

Fig 2M
anti-miR-145
anti-NC
-92kDa β-catenin

## Slide 6
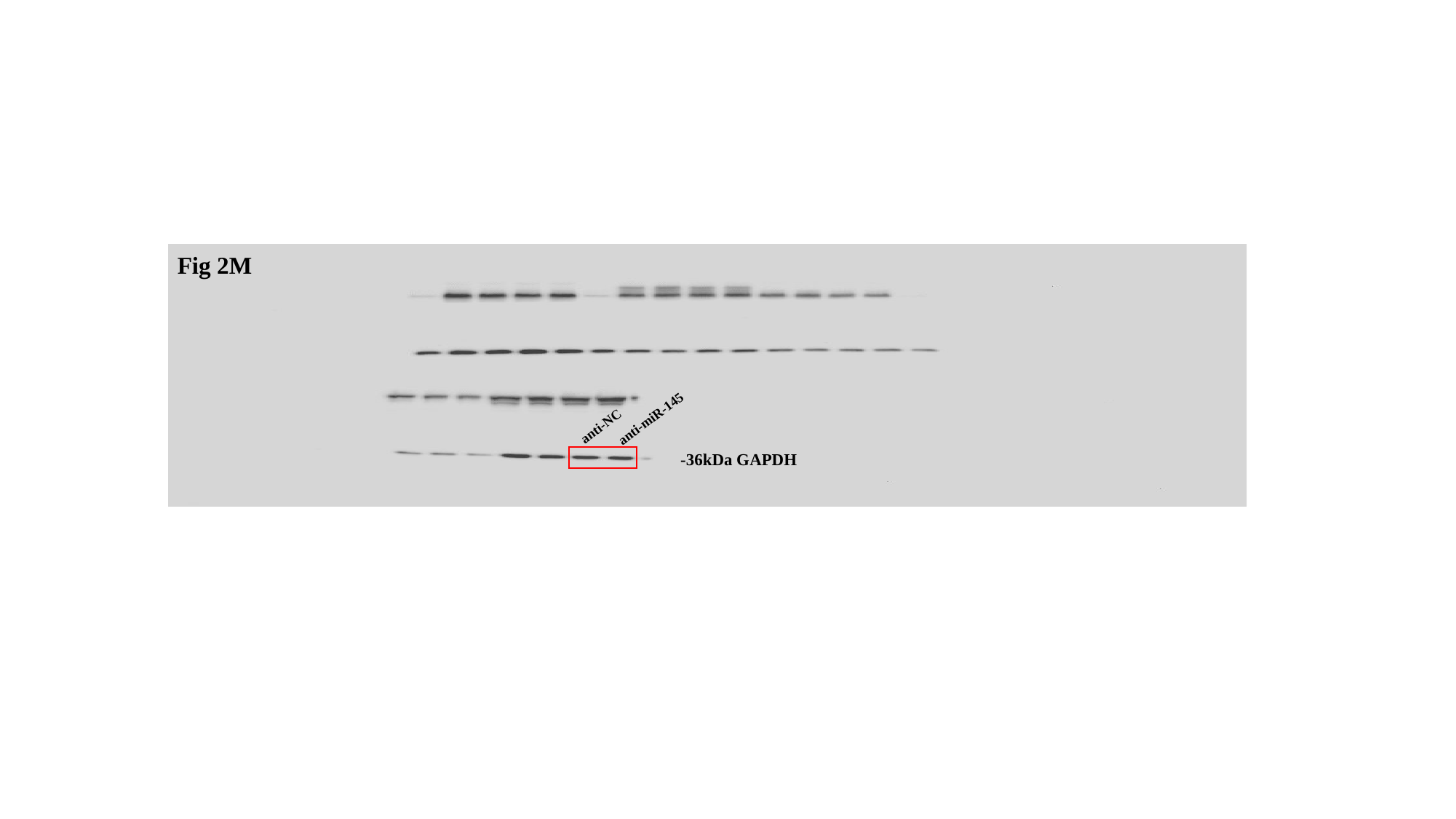

Fig 2M
anti-miR-145
anti-NC
-36kDa GAPDH

## Slide 7
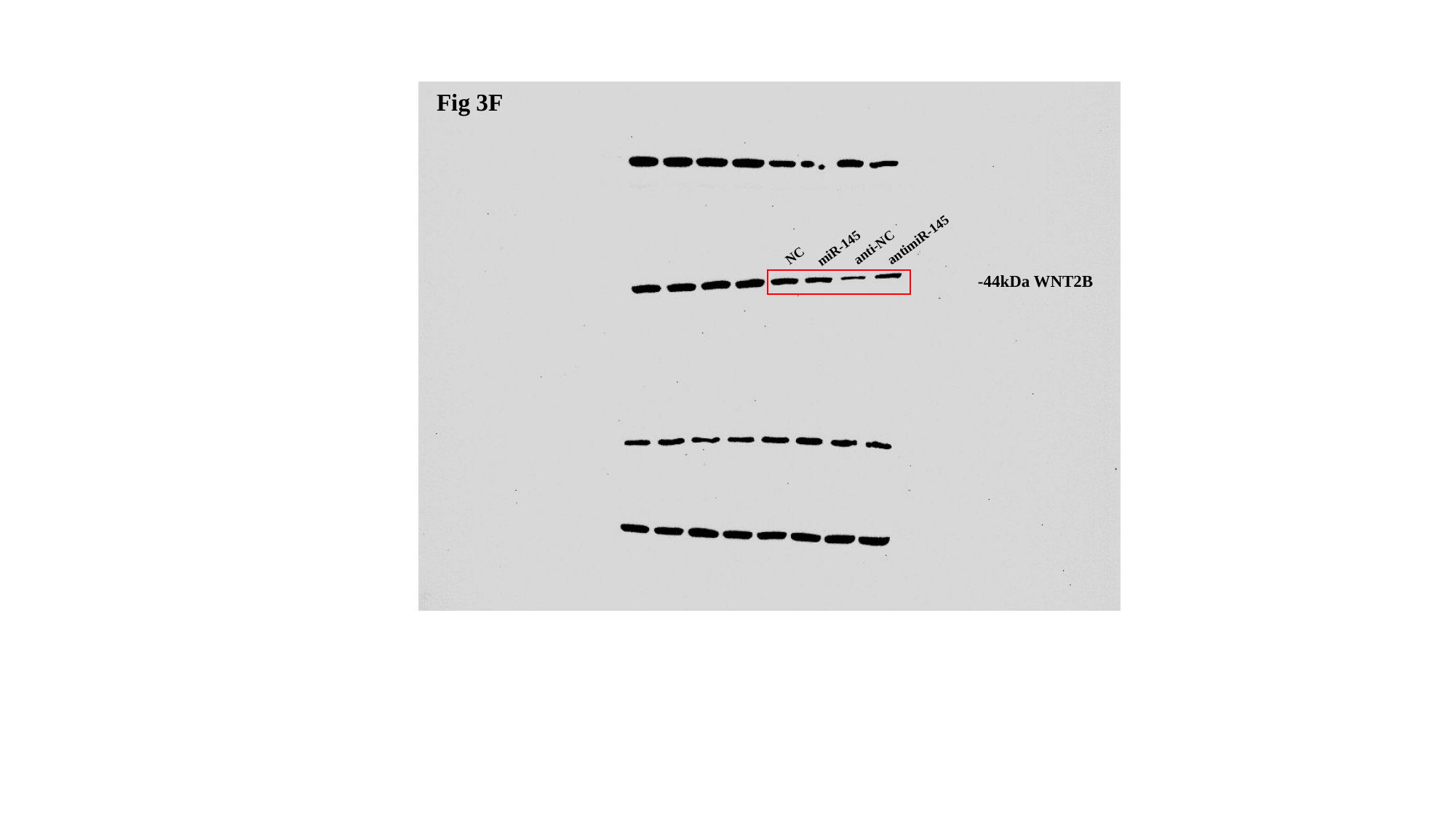

Fig 3F
antimiR-145
anti-NC
miR-145
NC
-44kDa WNT2B

## Slide 8
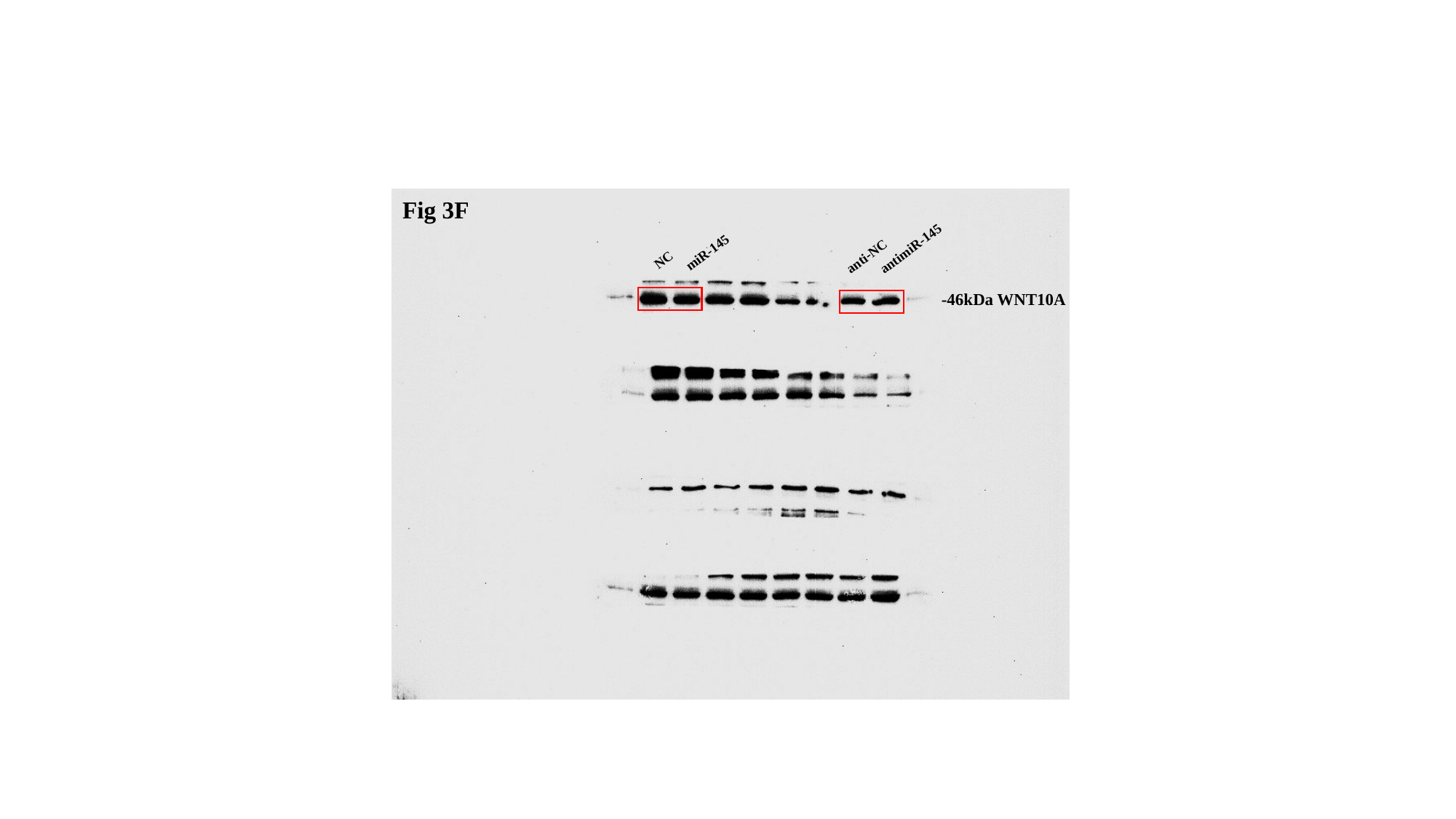

Fig 3F
antimiR-145
miR-145
anti-NC
NC
-46kDa WNT10A

## Slide 9
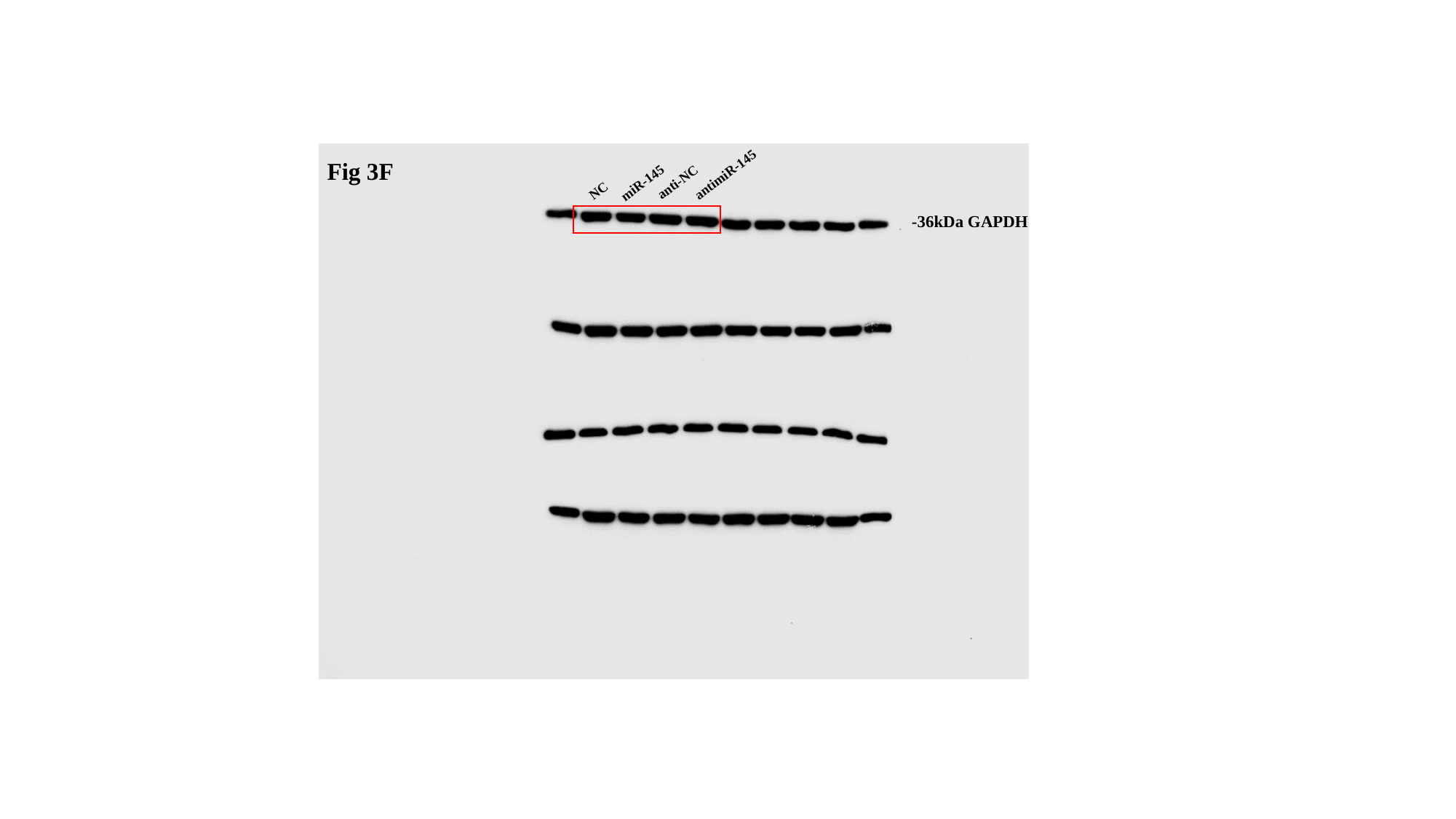

Fig 3F
antimiR-145
anti-NC
miR-145
NC
-36kDa GAPDH

## Slide 10
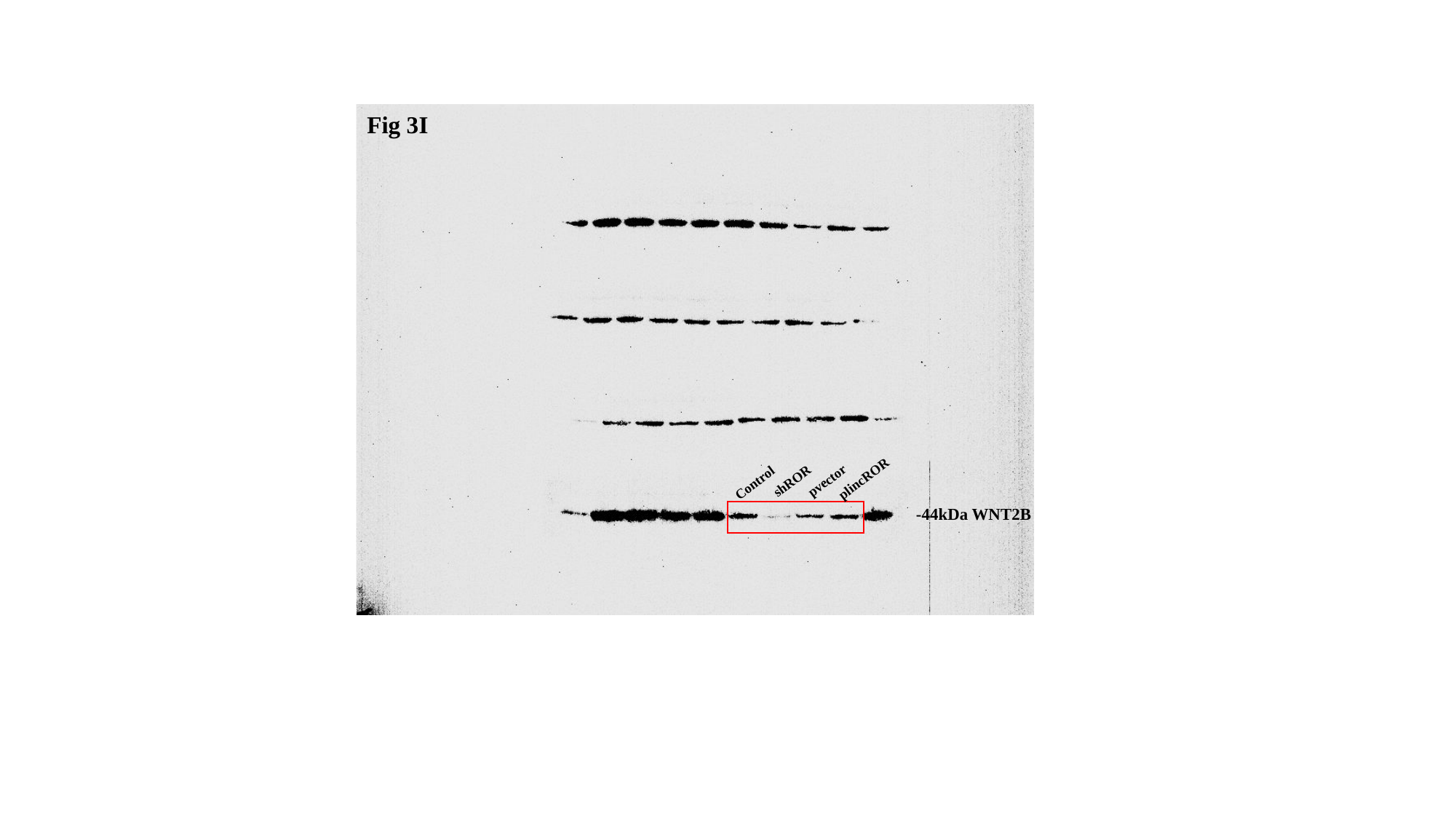

Fig 3I
plincROR
pvector
shROR
Control
-44kDa WNT2B

## Slide 11
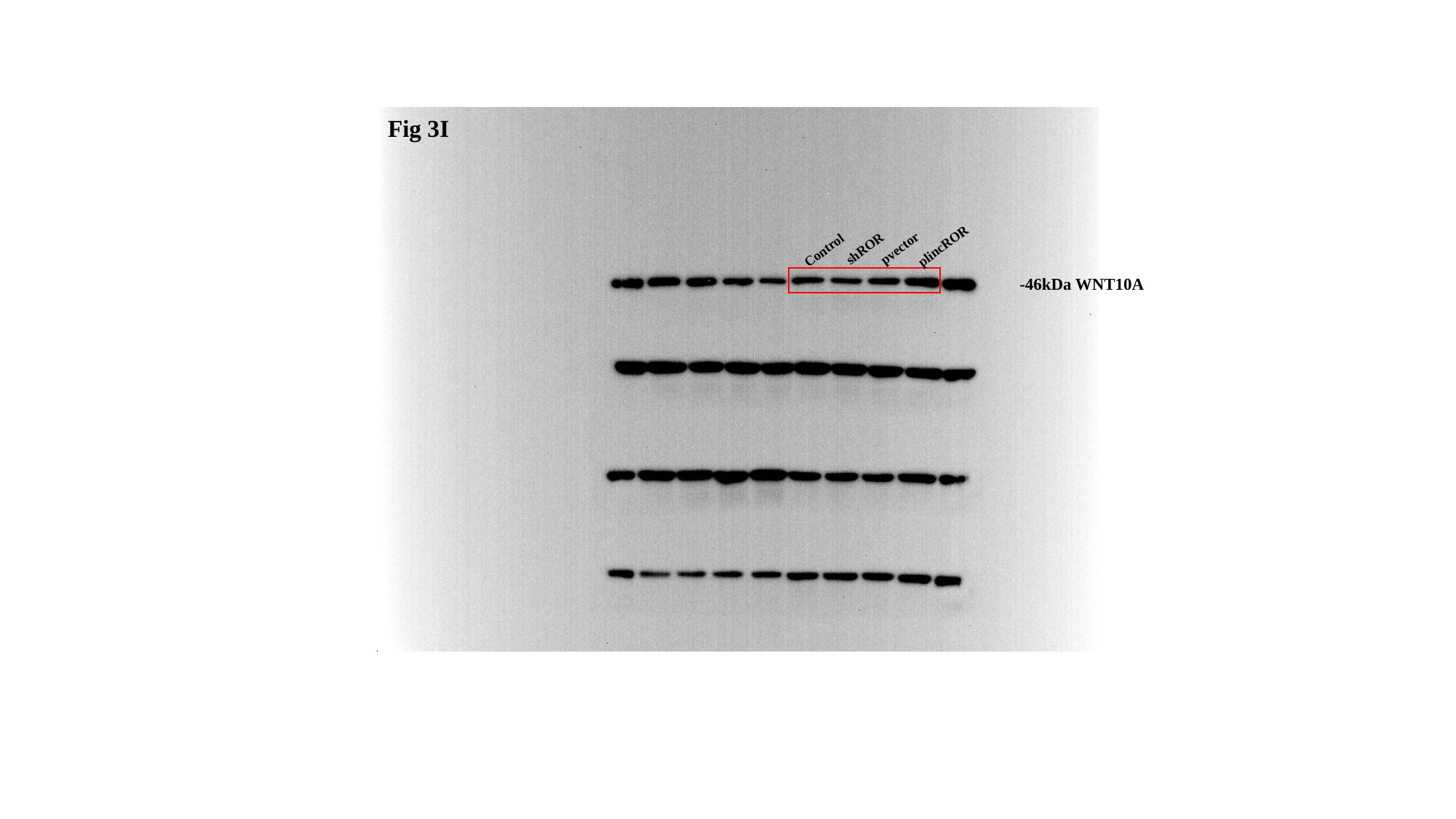

Fig 3I
plincROR
pvector
shROR
Control
-46kDa WNT10A

## Slide 12
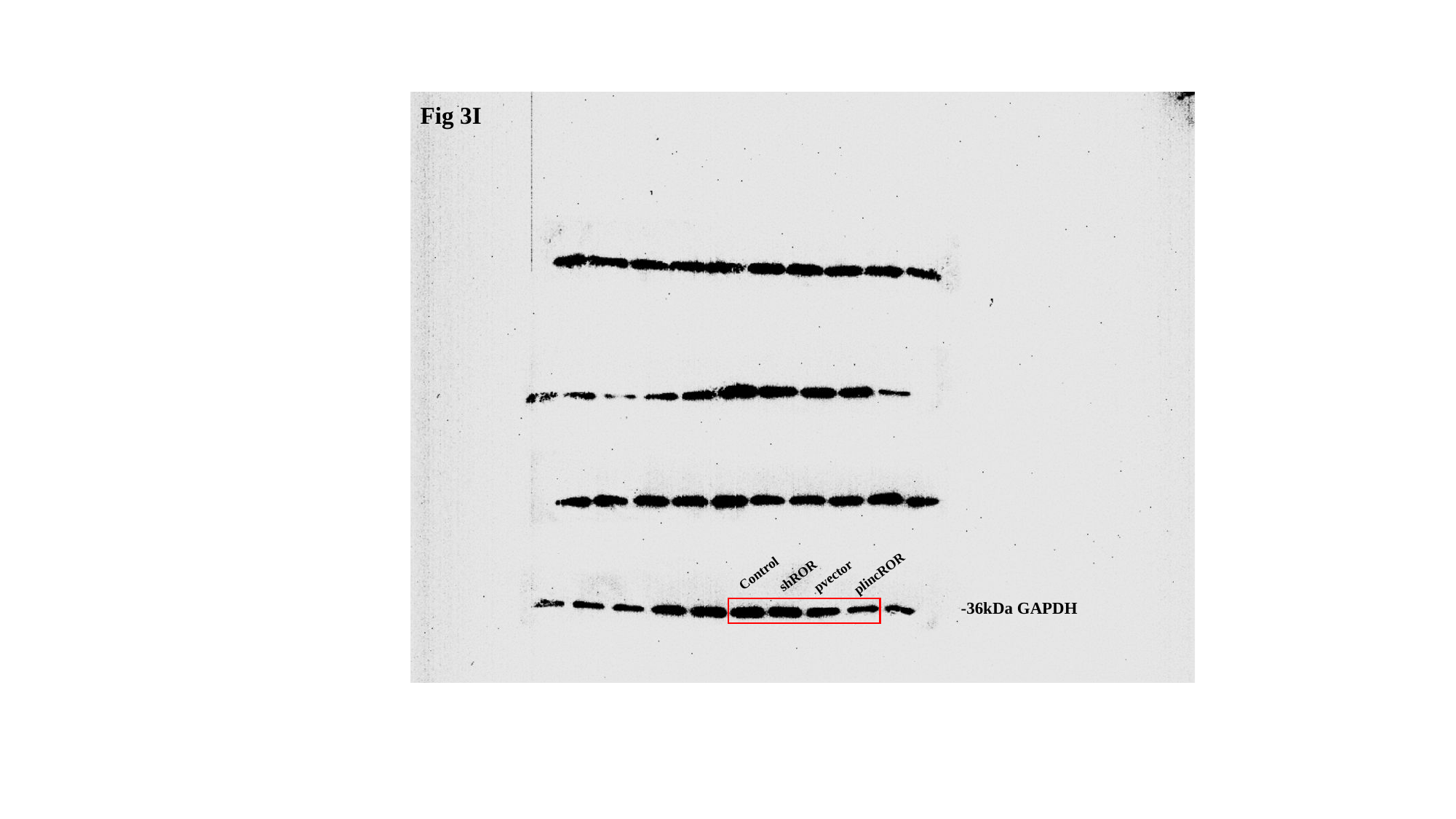

Fig 3I
Control
plincROR
shROR
pvector
-36kDa GAPDH

## Slide 13
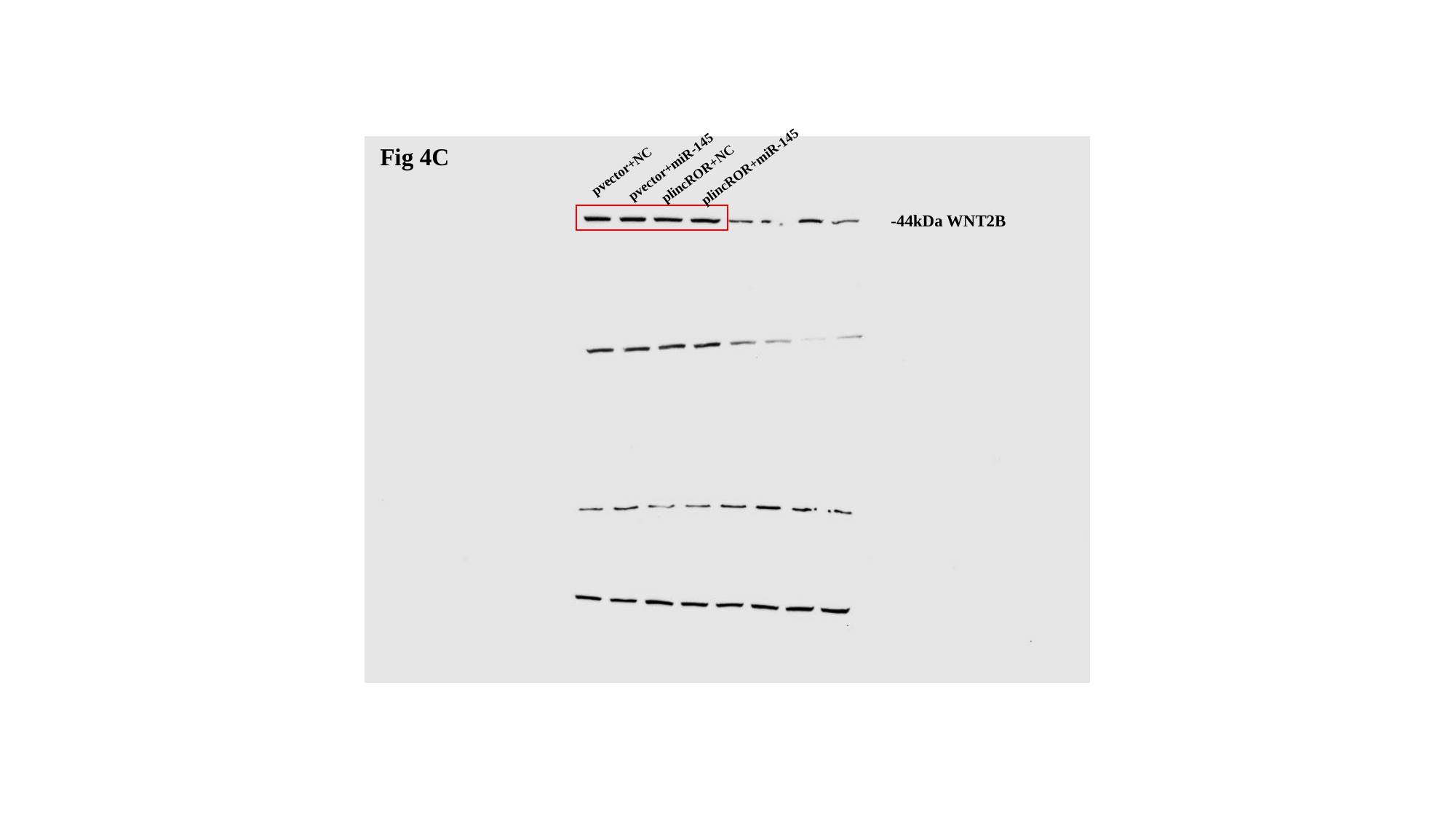

Fig 4C
pvector+miR-145
plincROR+miR-145
pvector+NC
plincROR+NC
-44kDa WNT2B

## Slide 14
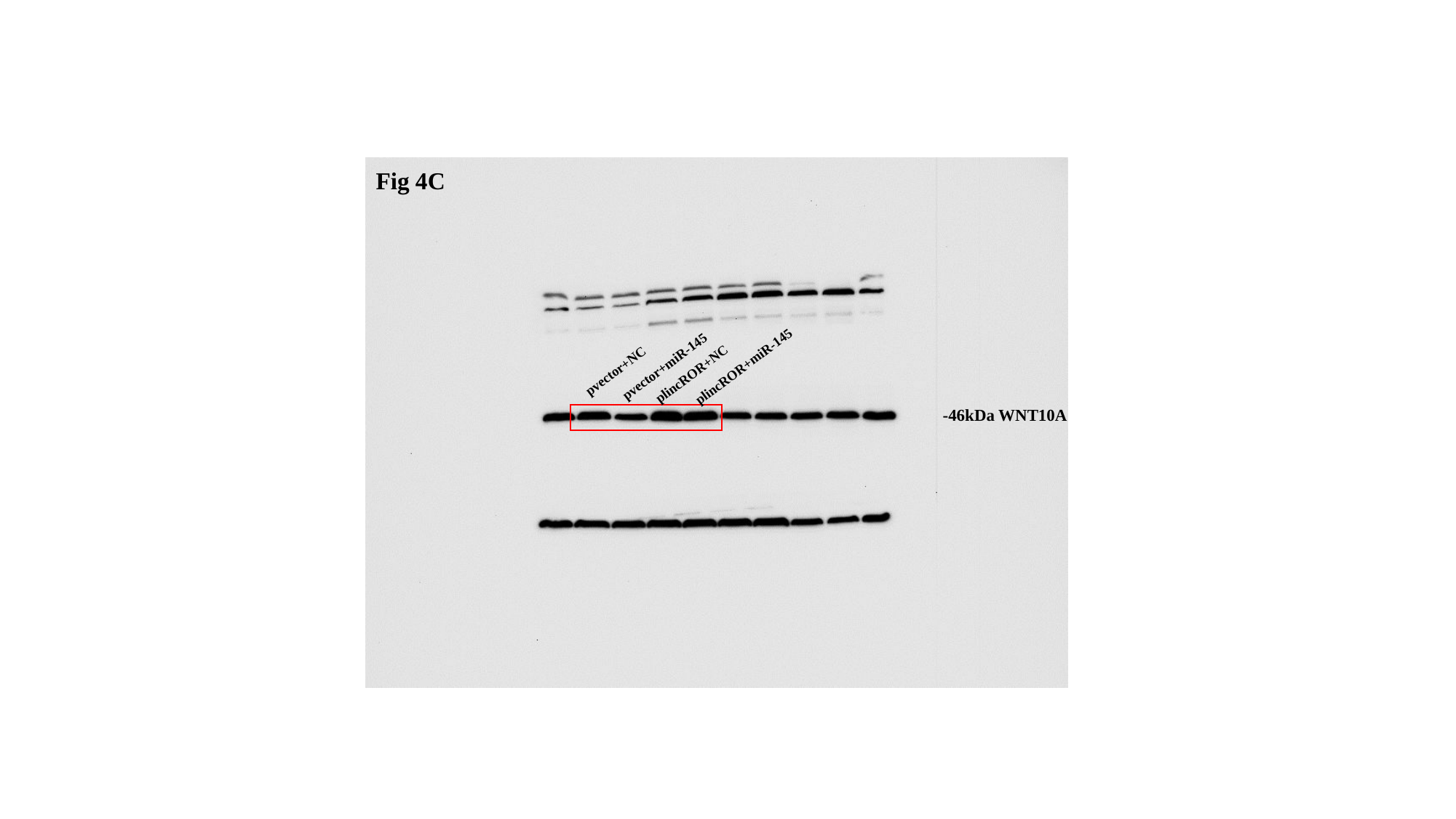

Fig 4C
pvector+miR-145
plincROR+miR-145
pvector+NC
plincROR+NC
-46kDa WNT10A

## Slide 15
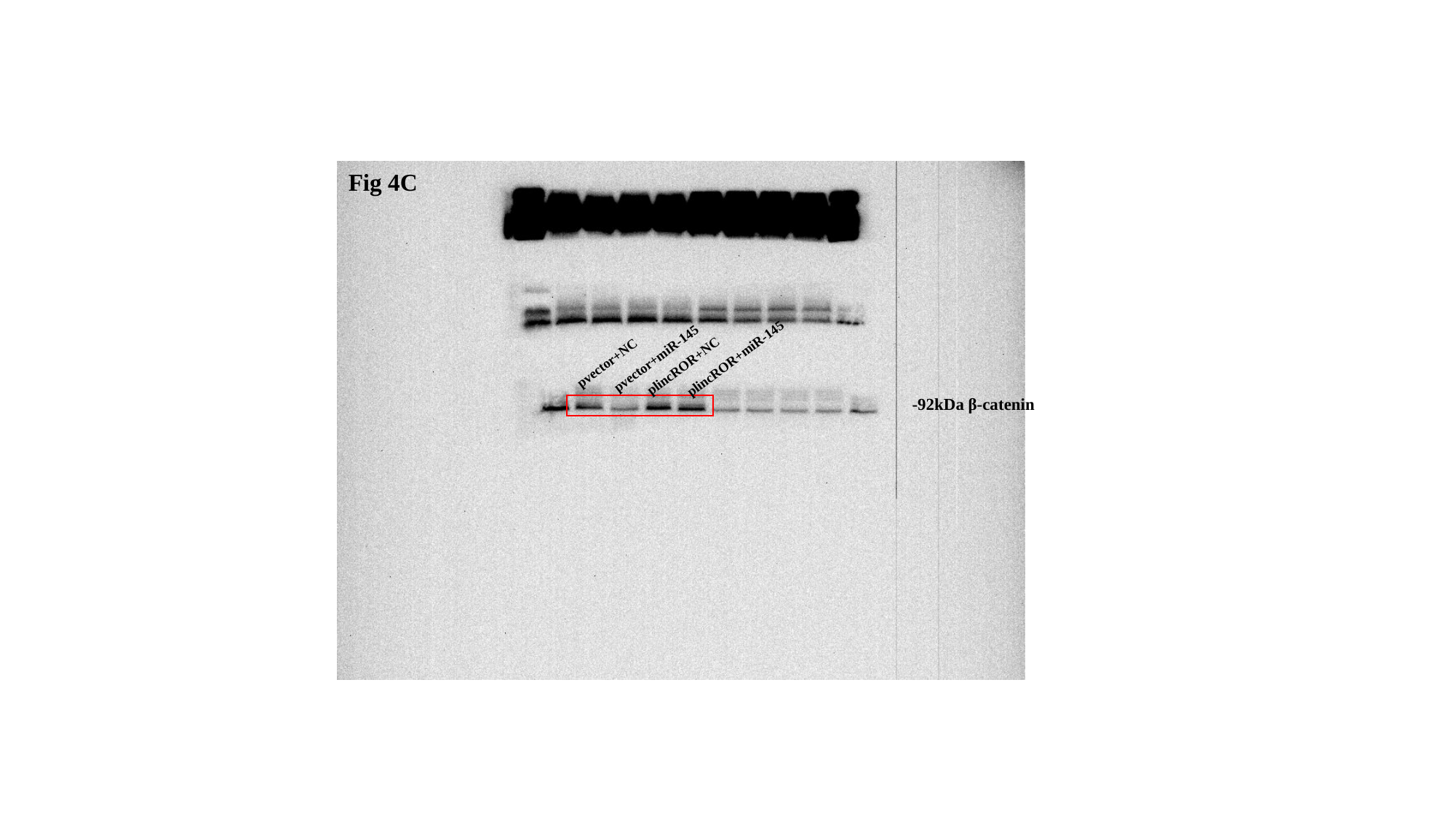

Fig 4C
pvector+miR-145
plincROR+miR-145
pvector+NC
plincROR+NC
-92kDa β-catenin

## Slide 16
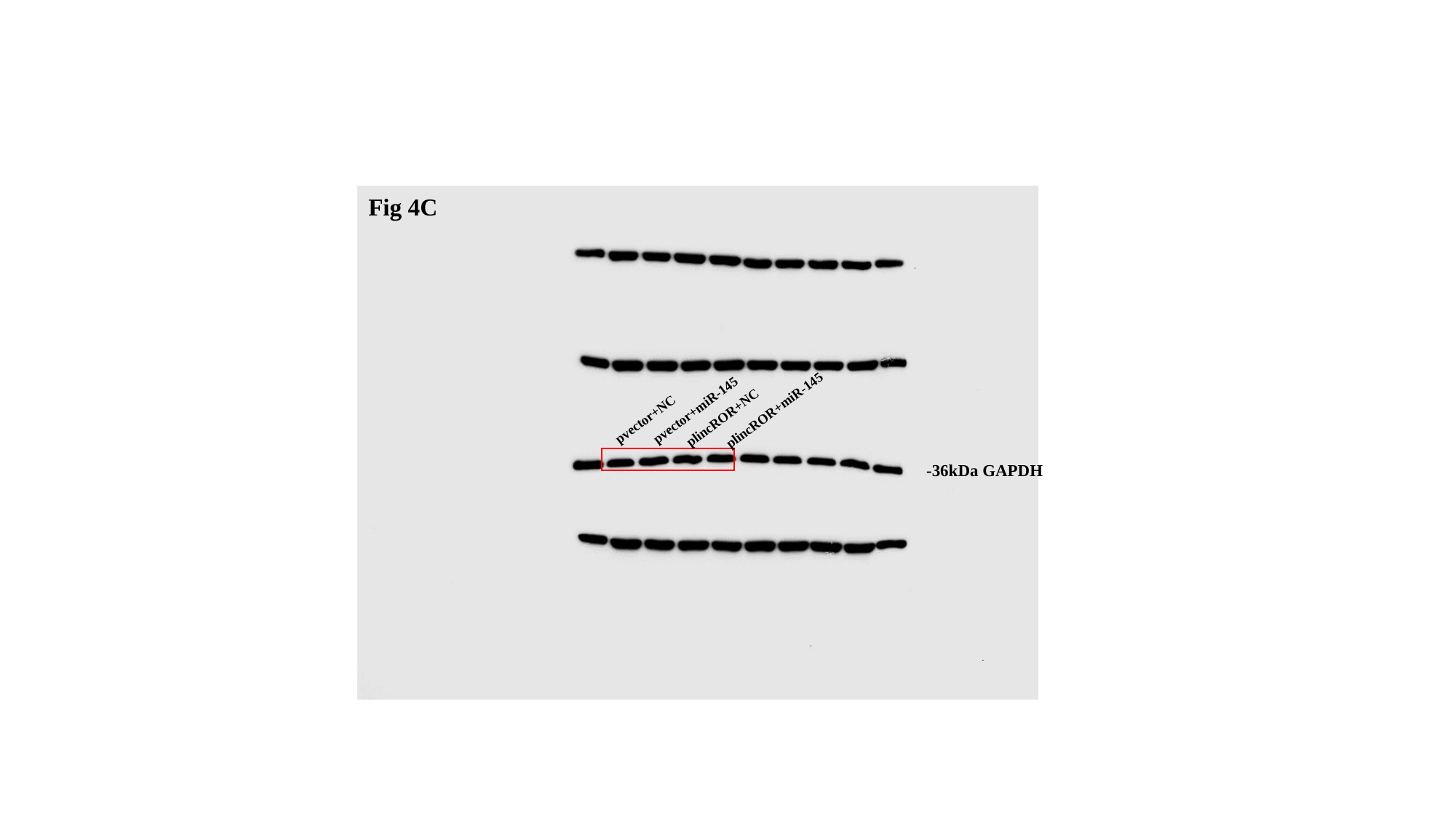

Fig 4C
plincROR+miR-145
pvector+miR-145
plincROR+NC
pvector+NC
-36kDa GAPDH

## Slide 17
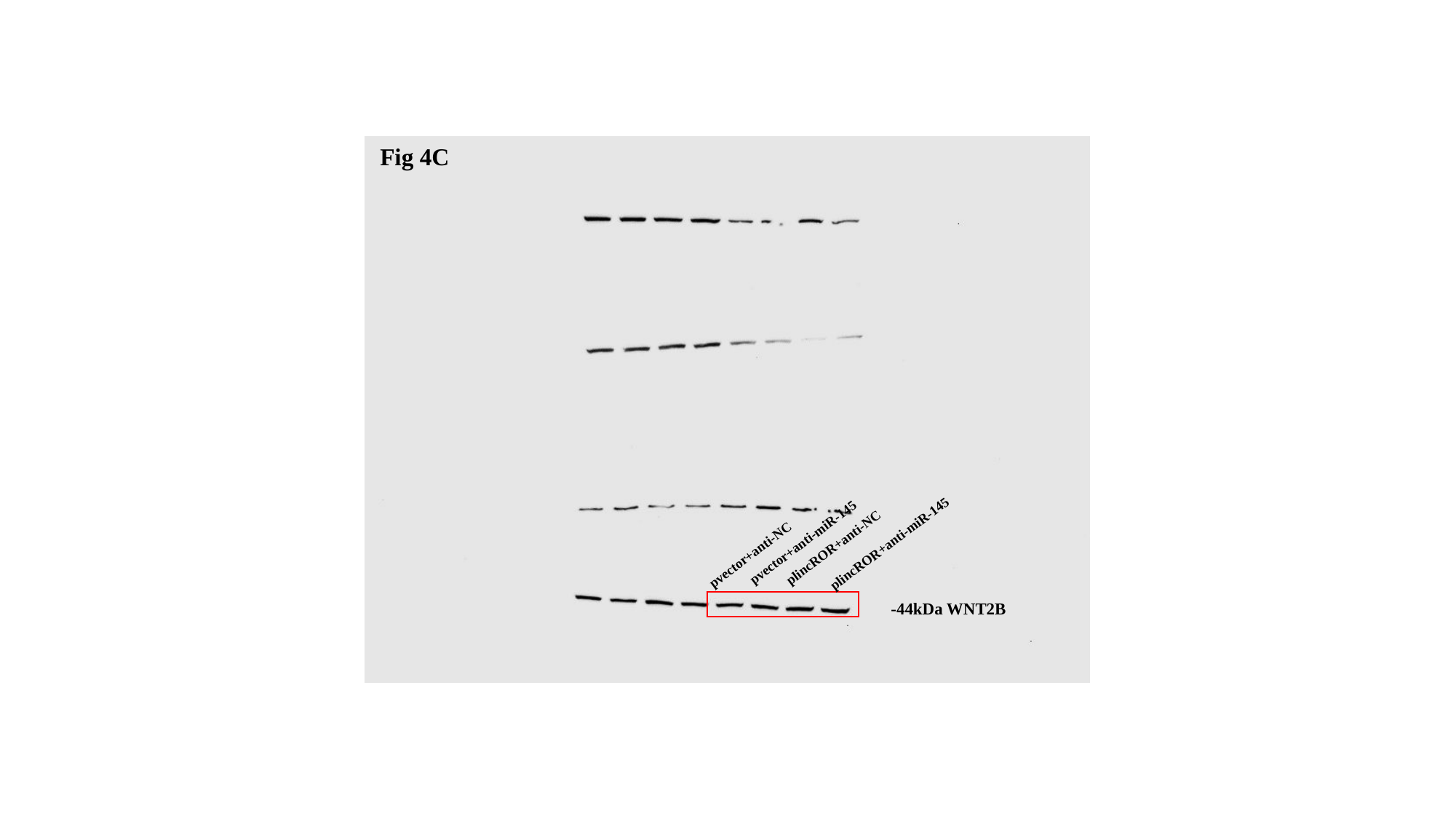

Fig 4C
pvector+anti-miR-145
plincROR+anti-miR-145
plincROR+anti-NC
pvector+anti-NC
-44kDa WNT2B

## Slide 18
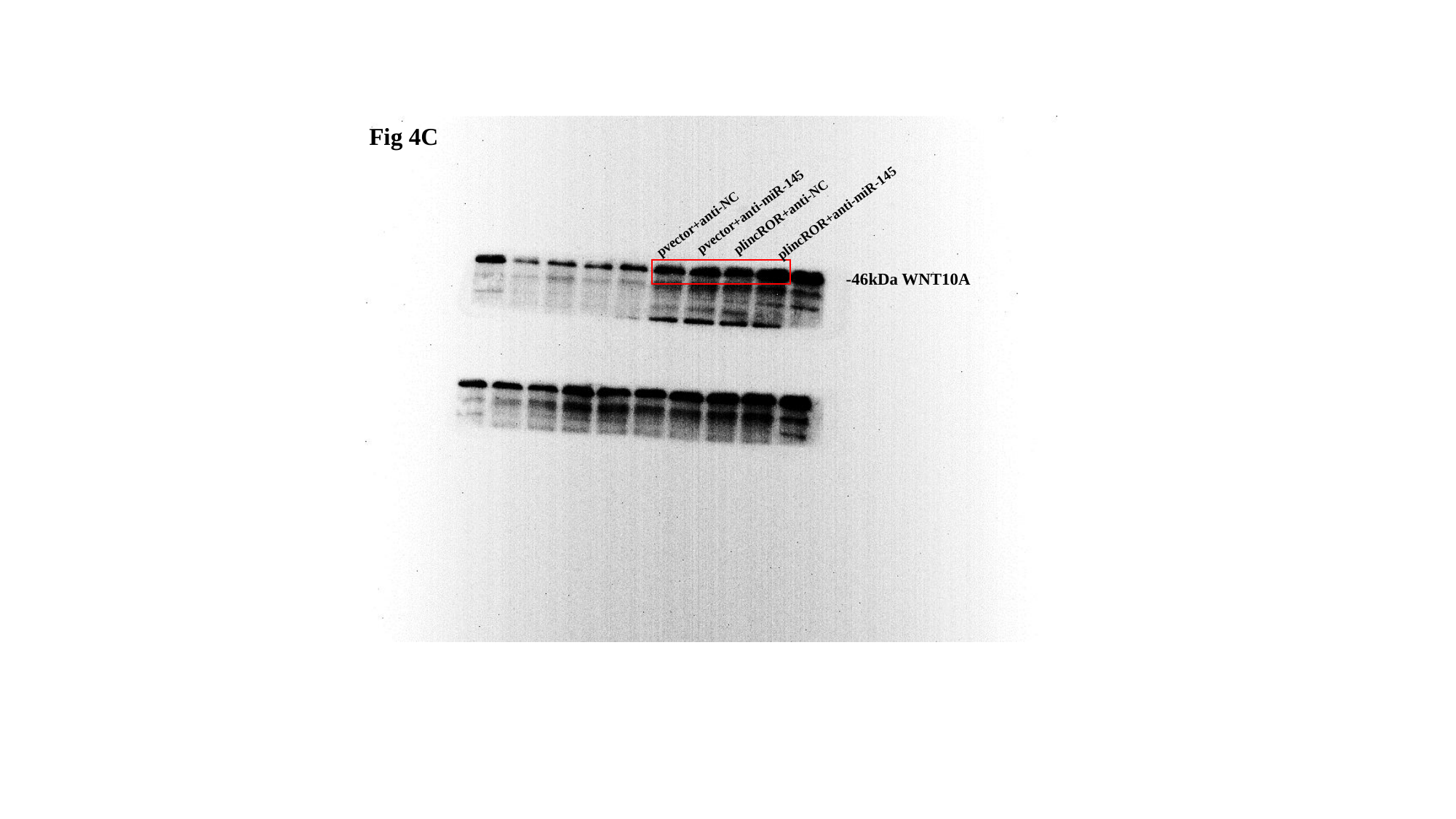

Fig 4C
pvector+anti-miR-145
plincROR+anti-miR-145
plincROR+anti-NC
pvector+anti-NC
-46kDa WNT10A

## Slide 19
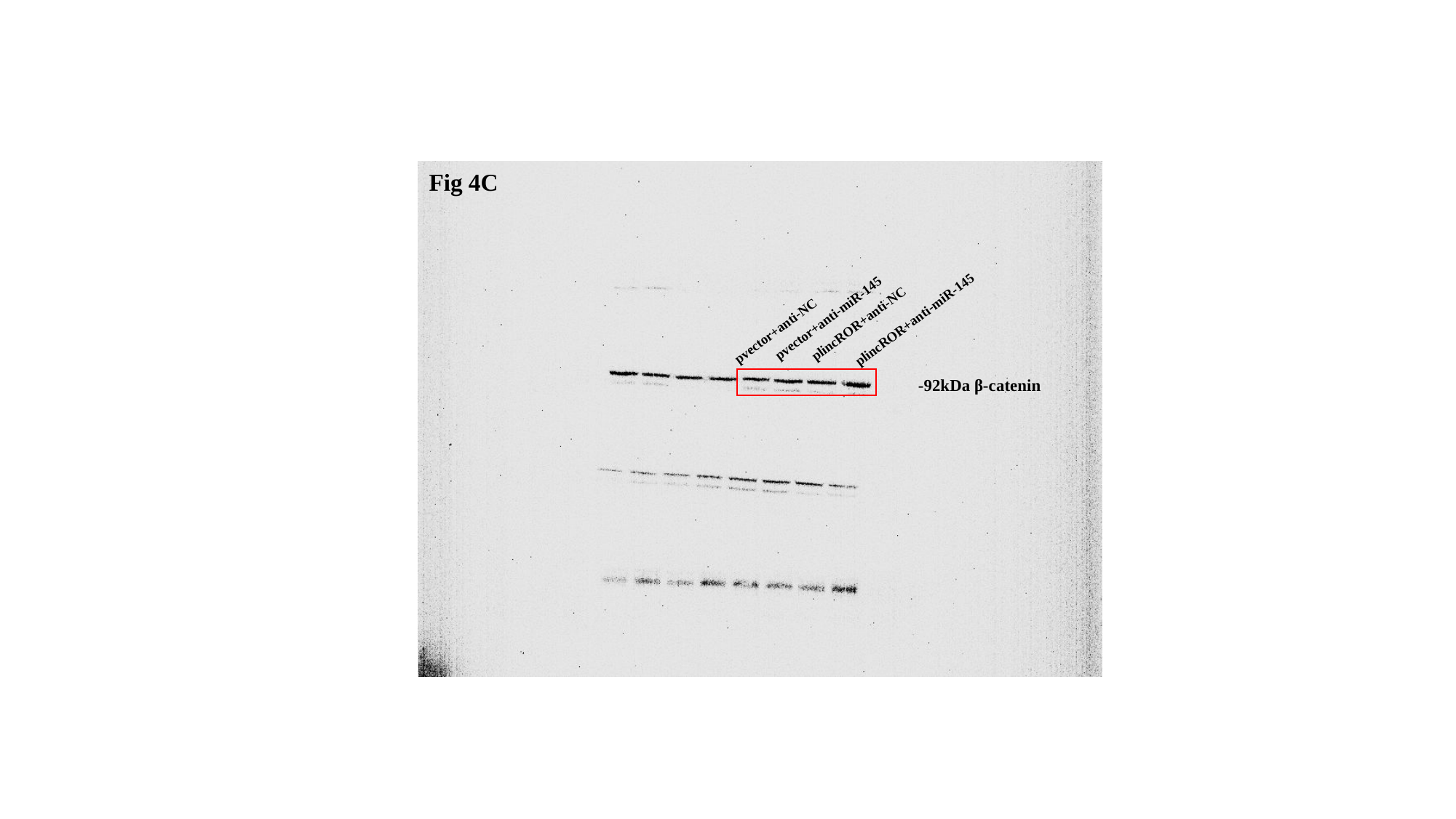

Fig 4C
pvector+anti-miR-145
plincROR+anti-miR-145
plincROR+anti-NC
pvector+anti-NC
-92kDa β-catenin

## Slide 20
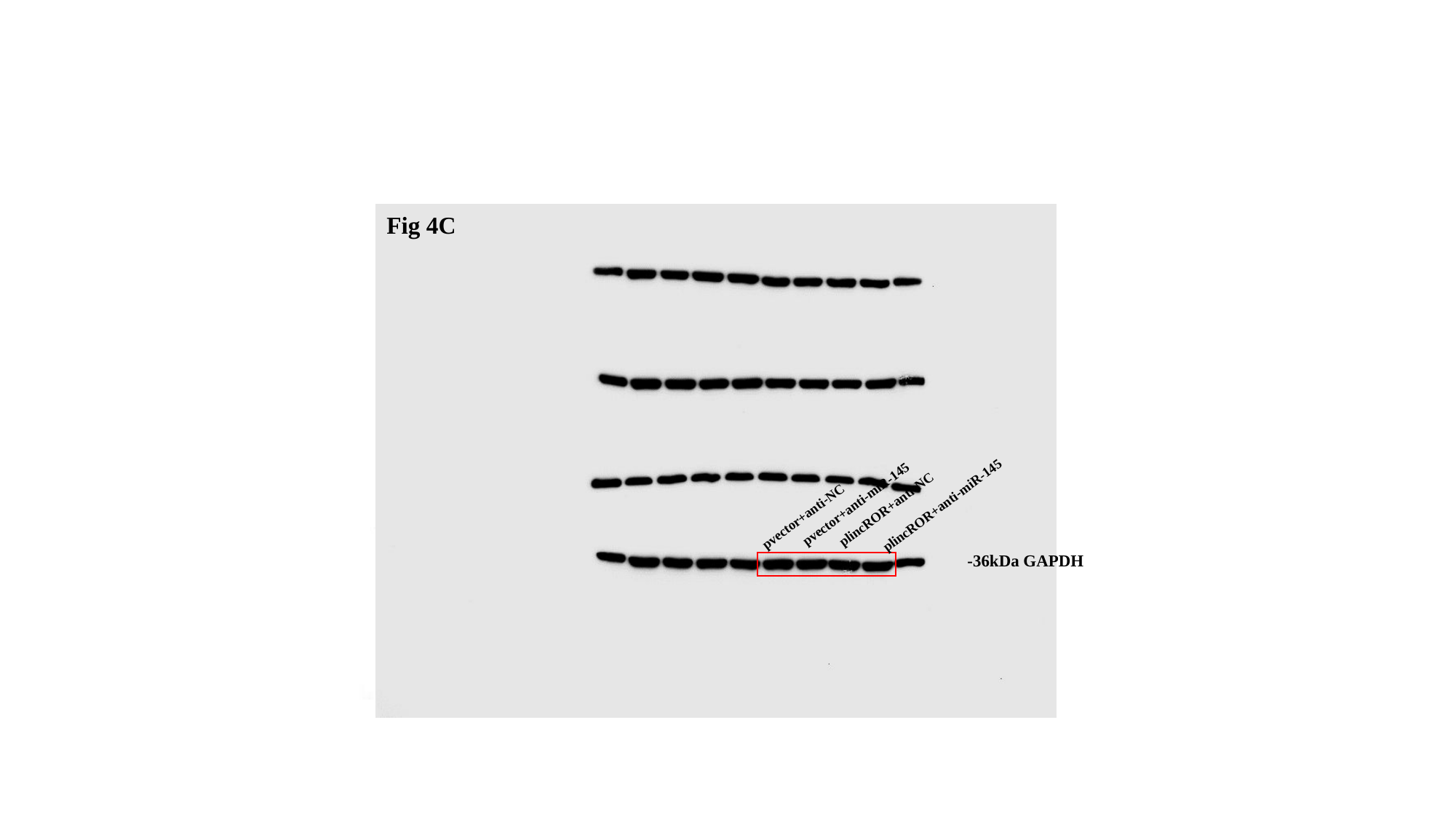

Fig 4C
pvector+anti-miR-145
plincROR+anti-miR-145
plincROR+anti-NC
pvector+anti-NC
-36kDa GAPDH
